# *In silico* Screening Unveil the Great Potential of Ruminal Bacteria Synthesizing Lasso Peptides

**DOI:** 10.3389/fmicb.2020.576738

**Published:** 2020-09-11

**Authors:** Yasmin Neves Vieira Sabino, Katialaine Corrêa de Araújo, Fábia Giovana do Val de Assis, Sofia Magalhães Moreira, Thaynara da Silva Lopes, Tiago Antônio de Oliveira Mendes, Sharon Ann Huws, Hilário C. Mantovani

**Affiliations:** ^1^Departamento de Microbiologia, Universidade Federal de Viçosa, Viçosa, Brazil; ^2^Departamento de Bioquímica e Biologia Molecular, Universidade Federal de Viçosa, Viçosa, Brazil; ^3^Institute for Global Food Security, School of Biological Sciences, Medical Biology Centre, Queen’s University Belfast, Belfast, United Kingdom

**Keywords:** precursor sequence, RiPPs, rumen, *Butyrivibrio*, antiSMASH 5, BAGEL4

## Abstract

Studies of rumen microbial ecology suggest that the capacity to produce antimicrobial peptides could be a useful trait in species competing for ecological niches in the ruminal ecosystem. However, little is known about the synthesis of lasso peptides by ruminal microorganisms. Here we analyzed the distribution and diversity of lasso peptide gene clusters in 425 bacterial genomes from the rumen ecosystem. Genome mining was performed using antiSMASH 5, BAGEL4, and a database of well-known precursor sequences. The genomic context of the biosynthetic clusters was investigated to identify putative *lasA* genes and protein sequences from enzymes of the biosynthetic machinery were evaluated to identify conserved motifs. Metatranscriptome analysis evaluated the expression of the biosynthetic genes in the rumen microbiome. Several incomplete (*n* = 23) and complete (*n* = 11) putative lasso peptide clusters were detected in the genomes of ruminal bacteria. The complete gene clusters were exclusively found within the phylum *Firmicutes*, mainly (48%) in strains of the genus *Butyrivibrio*. The analysis of the genetic organization of complete putative lasso peptide clusters revealed the presence of co-occurring genes, including kinases (85%), transcriptional regulators (49%), and glycosyltransferases (36%). Moreover, a conserved pattern of cluster organization was detected between strains of the same genus/species. The maturation enzymes LasB, LasC, and LasD showed regions highly conserved, including the presence of a transglutaminase core in LasB, an asparagine synthetase domain in LasC, and an ABC-type transporter system in LasD. Phylogenetic trees of the essential biosynthetic proteins revealed that sequences split into monophyletic groups according to their shared single common ancestor. Metatranscriptome analyses indicated the expression of the lasso peptides biosynthetic genes within the active rumen microbiota. Overall, our *in silico* screening allowed the discovery of novel biosynthetic gene clusters in the genomes of ruminal bacteria and revealed several strains with the genetic potential to synthesize lasso peptides, suggesting that the ruminal microbiota represents a potential source of these promising peptides.

## Introduction

Natural products have improved human quality of life and play a noteworthy role in drug discovery and development (Newman and Cragg, 2016). Among natural products, secondary metabolites stand out as scaffolds for the development of products for human medicine, animal health, crop protection, and numerous biotechnological applications (Bachmann et al., 2014). Traditional culture-based strategies for the screening of new molecules have been responsible for the discovery of many relevant enzymes and metabolites (Steele and Stowers, 1991; Winter et al., 2011). However, these approaches are largely driven by chance, making then costly, time-consuming, and often limited regarding the number of strains that can be used in large-scale screening endeavors (Tietz et al., 2017). The advent of microbial genomics and the increasing availability of computational tools to perform genome mining has evidenced the underexplored potential of some microbial species as alternative sources of new therapeutic agents (Winter et al., 2011). These tools and resources emerged as an alternative approach to identify novel biosynthetic gene clusters (BGCs) encoding putative bioactive metabolites and to assess the genetic potential of producer strains (Weber and Kim, 2016). Besides the discovery of new products, genome mining also contributes to understanding the connection between metabolites and the gene sequences that encode them, providing ecological insights about the role of individual microbial populations in the microbiome (Bachmann et al., 2014).

Secondary metabolites could play a diverse role in the environment by their wide range of biological activities. In this context, lasso peptides stand out as functionally diverse metabolites produced by several species of bacteria. Members of the lasso peptide family are reported to have antimicrobial (Salomon and Farías, 1992; Kuznedelov et al., 2011) and anti-viral activities (Frechet et al., 1994; Constantine et al., 1995). These molecules also can show receptor antagonism activities, such as the glucagon receptor antagonist BI-32169 (Potterat et al., 2004; Knappe et al., 2010) or act as enzyme inhibitors (Katahira et al., 1996; Yano et al., 1996). This functional diversity combined with their physicochemical properties makes lasso peptides attractive scaffolds for drug development (Knappe et al., 2011). Moreover, these molecules compose a class of ribosomally synthesized and post-translationally modified peptides (RiPPs), which are suitable for genome mining approaches due to the gene-encoded nature of their precursors (Maksimov and Link, 2014).

The lasso peptides typically contain 16–21 amino acid residues and are defined by their unusual topology, which resembles threaded lassos or slipknots. This peculiar structure is the result of cyclization, which is due to the amide bond between the amino group of an N-terminal Gly/Cys residue and the carboxyl group of a Glu/Asp residue at position 8 or 9 of the mature peptide (Bayro et al., 2003; Rosengren et al., 2003; Maksimov et al., 2012a). This post-translational modification is performed by a protease that shows homology to bacterial transglutaminases (LasB) and by a protein homologous to asparagine synthetase (LasC) (Duquesne et al., 2007a; Severinov et al., 2007). It is assumed that LasC is involved in the activation of the side-chain carboxyl group of the Glu/Asp residue at position 8 or 9, while LasB catalyzes the transfer of ammonia from glutamine to the activated side-chain carboxyl group. Following this, the cleavage of the precursor peptide releases an N-terminal Gly/Cys, and the cyclization takes place by a nucleophilic attack (Larsen et al., 1999; Makarova et al., 1999; Duquesne et al., 2007a; Yan et al., 2012). The gene D is also frequently found on the biosynthetic gene clusters of lasso peptides encoding an ABC transporter that is thought to play a role in immunity of producer cells against the antimicrobial activity of their lasso peptides (Solbiati et al., 1996, 1999; Bountra et al., 2017).

Studies based on genome mining have contributed to identifying new microbial species producing lasso peptides (Knappe et al., 2008; Maksimov et al., 2012b; Tietz et al., 2017). The ruminal ecosystem is composed of microbial communities that show high taxonomic and functional diversity (Morais and Mizrahi, 2019), and although it has been investigated as a source for novel enzymes and antimicrobials (Oyama et al., 2017; Neumann and Suen, 2018; Palevich et al., 2019), the rumen still represents an underexplored environment for the discovery of lasso peptides. Indeed, both culture-dependent and culture-independent approaches have revealed promising antimicrobial peptides from the rumen (Mantovani et al., 2002; Russell and Mantovani, 2002; Azevedo et al., 2015; Oyama et al., 2017, 2019). However, no systematic efforts have been made to investigate the potential of rumen bacteria to produce lasso peptides. The availability of hundreds of reference genomes from cultured ruminal bacteria through the Hungate1000 Project^1^, offers an unprecedented opportunity to identify novel lasso peptides within the genomes of ruminal bacteria. The present study set out to perform an *in silico* screening of the genomes of the major bacterial species represented in the core ruminal microbiome in an attempt to identify biosynthetic gene clusters encoding putative lasso peptides. As such, this work aimed to *i*) characterize the distribution of BGCs potentially associated with the production of lasso peptides in the genomes of ruminal bacteria; *ii*) identify sequences of potential novel lasso peptide precursors, *iii*) evaluate the phylogenetic distribution and conservation of the biosynthetic genes/proteins predicted to encode lasso peptides, and *iv*) examine if these biosynthetic genes are expressed by the active rumen microbiota.

## Materials and Methods

### Genomic Data Collection and Prediction of Lasso Peptides Within Rumen Bacterial Genomes

Genome files (.fasta) of 425 ruminal bacteria belonging to the Hungate1000 project (Seshadri et al., 2018) were downloaded from the NCBI (National Center for Biotechnology Information^2^ and JGI (Joint Genome Institute^3^) websites (Supplementary Table 1).

To identify microcin producers, the amino acid sequences of McjA (AAD28494.1), McjB (AAD28495.1), McjC (AGC14226.1), and McjD (AGC14214.1), which are products of the *mcj*ABCD gene cluster, were downloaded from NCBI. A set of 68 lasso peptides previously described in the literature were also screened based on their core sequences (Zyubko et al., 2019) (Supplementary Table 2). Screening of these protein-coding genes in genomes of ruminal bacteria was performed by running BLASTx through the command line. The cut-off parameters used to consider positive hits were a minimum sequence identity of 30% and *E*-value < 10^–5^. New putative biosynthetic gene clusters in the genomes of ruminal bacteria were predicted using the web-based genome mining tools of antiSMASH 5 (Blin et al., 2019) and BAGEL4 (van Heel et al., 2018).

The genes belonging to predicted clusters were generically named *lasA*, *lasB*, *lasC*, and *lasD*, corresponding to the following putative gene products: LasA, the lasso peptide precursor; LasB, the leader peptidase, LasC, the lasso cyclase and LasD, the ABC transporter (Arnison et al., 2013).

### Distribution of BGCs Encoding Putative Lasso Peptides in the Genomes of Ruminal Bacteria

To verify the distribution of biosynthetic gene clusters encoding putative lasso peptides in the genomes of rumen bacteria, a phylogenetic tree was reconstructed using the 16S rRNA gene sequences from the rumen bacterial genomes analyzed in this study. The sequences were obtained from the Hungate1000 Project and aligned using RDP Release 11.5 aligner of the Ribosomal Database Project website (RDP^4^). The phylogenetic tree was reconstructed using the Approximately Maximum-Likelihood method by FastTree v2.1 (Price et al., 2010). The Interactive Tree of Life (iTOL) interface v4^5^ (Letunic and Bork, 2019) was used to visualize and annotate the FastTree output file (.tree).

### Genomic Context of the Lasso Peptide Biosynthetic Gene Clusters

When a putative biosynthetic gene cluster was predicted by BAGEL4 and/or antiSMASH 5 but the gene encoding the potential precursor peptide was not identified, the regions located upstream and downstream of the predicted biosynthetic genes were examined, covering a region varying from 4762 bp to 17083 bp in length. This analysis is based on the assumption that genes sharing similar occurrence patterns or located near each other within the genome are likely to be functionally related. For that, genomes were annotated using Prokka v1.12 (Seemann, 2014) (minimum contig size of 200 kb and *E*-value < 10^–6^) through the Galaxy platform and manually inspected to identify sequences potentially encoding the lasso peptide precursor within the genomic context of the biosynthetic machinery minimally required for the production of lasso peptides. A manual analysis of the genes adjacent to the lasso peptide gene cluster was also performed to investigate the presence of co-occurring genes. When proteins were annotated as “hypothetical”, BLAST analyses were performed in Uniprot to investigate the protein function using 30% of amino acid sequence similarity as the cut-off parameter.

### Characterization of the Biosynthetic Proteins

#### Conservation Analysis of LasA, LasB, and LasC

Conserved motifs in putative lasso peptide precursors (LasA) and maturation enzymes (LasB, and LasC) were identified using the Batch Web CD-Search tool (Lu et al., 2020). Default parameters were used for the analysis and the results were filtered considering *E*-value < 10^–6^. To analyze if the amino acid sequences of the biosynthetic precursors/proteins (LasA, LasB, and LasC) could be grouped according to the evolutionary history of the major species of bacteria from the rumen microbiome, phylogenetic trees were reconstructed based on the sequences of these proteins. The sequences were aligned using muscle 3.8.31. FastTree v2.1 was used to perform the tree reconstruction and iTOL was used to visualize and annotate the tree as described above. For LasA, the amino acid composition of conserved residues corresponding to the 44 amino acid residues located in the N-terminus of the protein was represented using WebLogo 2.8.2^6^ and the default parameters.

#### Prediction of the LasA Core

Predictions of putative LasA core sequences followed patterns of the distribution of amino acid residues that have been extensively reported in the literature for precursor and mature lasso peptide sequences (Jia Pan et al., 2012; Maksimov et al., 2012a,b; Tietz et al., 2017). The core sequences of the putative lasso peptide precursors were inferred using BAGEL4, antiSMASH 5, and manual analyses of the genomic sequences flanking the putative gene clusters. The presence of glycine (G) at position 1 of the core peptide, followed by an aspartate (D) located at position 8 or 9 were used as criteria indicative of the N-terminal macrolactam ring matching the lasso peptides precursor pattern. The presence of threonine (T) in the leader peptide near the cleavage site was also taken into account, as well as the presence of conserved amino acids at particular positions in the precursor peptides, as indicated by the alignment of putative LasA sequences. All sequences predicted as a putative core of LasA were run through RiPPMiner^7^ to confirm cross-links between post-translationally modified residues associated with the formation of a characteristic ring structure in lasso peptides (Agrawal et al., 2017).

### Expression of Lasso Peptide Genes in Rumen Metatranscriptomes

The expression of the genes *lasA, lasB*, and *lasC* predicted in the genomes of ruminal bacteria were investigated using different metatranscriptome datasets from the rumen. Sequences of fifteen metatranscriptomes were obtained from the sequence read archive (SRA) of NCBI^8^ (Leinonen et al., 2011). These datasets included ruminal metatranscriptomes from dairy and beef cattle and sheep, as described in Supplementary Table 3.

To evaluate the expression of unique *lasA*, *lasB*, and *lasC* genes, sequences corresponding to these genes but showing more than 50% similarities in a distance matrix calculated using Clustal Omega (Sievers et al., 2011) were eliminated from the downstream analyses. Bowtie-build tool was then used to index the lasso peptide sequences and Bowtie2/2.2.8 (Langmead and Salzberg, 2012) was applied to align the genes and the metatranscriptome datasets. The alignment results were visualized through Tablet software 1.19.09.03 (Milne et al., 2012). The expression levels of *lasA*, *lasB*, and *lasC* were normalized calculating the number of reads per kilobase per million of mapped reads (RPKM) (Mortazavi et al., 2008).

## Results

### Distribution of Predicted Biosynthetic Gene Clusters of Lasso Peptides in the Species of Ruminal Bacteria

In the current study, we performed data mining in 425 bacterial genomes representing the major species of bacteria from the rumen microbiome in an attempt to identify BGCs potentially associated with the production of lasso peptides. Computational tools were applied to search for sequences in the rumen bacterial genomes with similarities to genes that are known to be associated with the biosynthesis of lasso peptides. *Butyrivibrio proteoclasticus* P18 and *Lachnospiraceae bacterium* NK4A144 harbored a gene encoding a product matching the McjC protein previously characterized in the *mcjABCD* operon (30% as a sequence similarity cut-off and *E*-value < 10^–5^), which is associated with the production of microcin J25. Moreover, mining the rumen bacterial genomes using the core regions of 68 lasso peptide precursors as query sequences revealed that *Actinomyces denticolens* PA and *Bacillus cereus* KPR-7A harbor sequences of core peptides highly similar (>70%) to Ssv-2083 and Paeninodim, respectively.

The BGCs predicted using BAGEL4 and antiSMASH 5 were divided into complete clusters, when containing all the genes considered to be essential (*lasA*, *lasB* and *lasC*) for the biosynthesis machinery required for lasso peptide production, and those which appear to be incomplete gene clusters, containing at least one (but not all) of the genes required for lasso peptide biosynthesis. The genome mining approaches revealed thirty-four ruminal bacterial genomes harboring incomplete or complete biosynthetic gene clusters potentially encoding putative lasso peptides (Table 1). The genomes with positive hits belonged mainly to the genera *Butyrivibrio*, of which 14 incomplete clusters and 3 complete clusters were identified out of 53 genomes analyzed. Members of the genus *Lachnospira* sp. (*n* = 5) harbored 3 incomplete and 2 complete biosynthetic gene clusters. Biosynthetic clusters containing all the essential genes for lasso peptide biosynthesis were also identified in the genomes of ruminal *Bacillus* sp. (*n* = 3), while other genera of ruminal bacteria, such as *Acetitomaculum*, *Actinomyces*, *Clostridium*, *Eubacterium*, and *Ruminococcus* presented clusters in lower abundance or the number of available genomes was too few to allow inferences about the abundance of putative lasso peptide gene clusters (Supplementary Figure S1).

**TABLE 1:** Ruminal bacterial genomes harboring genes and gene clusters encoding putative lasso peptides predicted by BAGEL4 and antiSMASH 5.

Genomes^1^	BAGEL4				antiSMASH 5			
	*lasA*	*lasB*	*lasC*	*lasD*	*lasA*	*lasB*	*lasC*	*lasD*
*Acetitomaculum ruminis* DSM 5522	0	0	1	1	0	1	1	1
*Actinomyces denticolens* PA	1	0	1	1	0	0	0	0
***Bacillus cereus* KPR-7A**	0	0	0	0	1	1	1	1
***Bacillus licheniformis* VTM3R78**	0	0	1	1	1	1	1	1
***Bacillus* sp. MB2021**	0	0	1	1	1	1	1	1
***Butyrivibrio fibrisolvens* AB2020**	1	0	1	0	0	1	1	1
*Butyrivibrio fibrisolvens* AR40	0	0	1	0	0	1	1	0
***Butyrivibrio fibrisolvens* DSM 3071**	0	0	1	0	1	1	1	1
*Butyrivibrio fibrisolvens* MD2001	0	0	0	1	0	1	1	1
*Butyrivibrio fibrisolvens* WTE3004	0	0	1	0	0	1	1	1
*Butyrivibrio fibrisolvens* YRB2005	0	0	1	0	0	1	1	1
*Butyrivibrio proteoclasticus* B316	0	0	1	1	0	1	1	1
*Butyrivibrio proteoclasticus* FD2007	0	0	1	1	0	1	1	1
*Butyrivibrio* sp. FC2001	0	0	1	0	0	1	1	0
*Butyrivibrio* sp. INlla14	0	0	0	0	0	1	1	1
*Butyrivibrio* sp. MC2021	0	0	1	1	0	1	1	1
*Butyrivibrio* sp. NC3005	0	0	1	1	0	1	1	1
***Butyrivibrio* sp. VCD2006**	0	0	1	1	1	1	1	0
*Butyrivibrio* sp. XBB1001	0	0	1	1	0	1	1	1
*Butyrivibrio* sp. XPD2006	0	0	1	0	0	1	1	1
*Butyrivibrio* sp. YAB3001	0	0	1	1	0	1	1	1
*Clostridium beijerinckii* HUN142	0	0	1	0	0	1	1	1
*Clostridium butyricum* AGR2140	0	0	1	1	0	1	1	1
*Eubacterium celullosolvens* LD2006	0	0	0	0	0	1	1	0
***Lachnospira multipara* D15d**	0	0	1	1	1	1	1	1
*Lachnospira multipara* LB2003	0	0	1	1	0	1	1	1
***Lachnospira multipara* MC2003**	0	0	1	1	1	1	1	1
***Lachnospira multipara* ATCC 19207**	0	0	0	0	1	1	1	1
*Lachnospira pectinoschiza* M83	0	0	1	1	0	1	1	1
*Lachnospiraceae bacterium* KH1P17	0	0	0	0	0	1	1	0
*Lachnospiraceae bacterium* MA2020	0	0	1	1	0	1	1	1
*Lachnospiraceae bacterium* YSD2013	0	0	1	0	0	1	1	0
***Ruminococcus albus* 8**	0	0	0	0	1	1	1	1
***Ruminococcus flavefaciens* ATCC 19208**	1	0	0	1	1	1	1	1

*^1^Genomes in bold harbored all the essential biosynthetic genes (*lasA, lasB, and lasC*) required for lasso peptide production detected by using either BAGEL4 or antiSMASH 5. Number 1 represents the presence of a gene while “0” represents the absence of a gene.*

**FIGURE 1:**
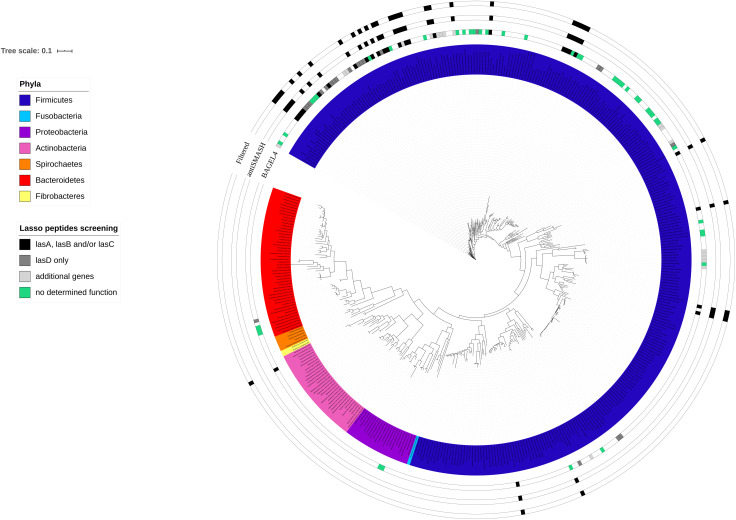
Distribution of predicted genes and gene clusters encoding putative lasso peptides in ruminal bacterial genomes superimposed to the 16S rRNA phylogenetic tree. The colored innermost circle represents the bacterial phyla analyzed in this study. Squared boxes outside the phylogenetic tree show the lasso peptide genes within the ruminal bacterial genomes predicted by BAGEL4 and antiSMASH 5. Filtered genomes contain at least one of the essential biosynthetic genes (*lasA, lasB*, and *lasC*) required for lasso peptide production. The sequences of the 16S rRNA gene were obtained from the Hungate1000 database. The alignment was performed in RDP and the tree was reconstructed using FastTree 2.1 (Maximum Likelihood, 1000 replicates). Features shown in the figure were added using iTOL annotation.

The number of genes and biosynthetic clusters that were predicted in the ruminal bacterial genomes varied according to the computational tool used to screen for the lasso peptides (Figure 1). In total, BAGEL4 predicted putative lasso peptide biosynthetic gene clusters in 109 ruminal bacterial genomes. However, the majority of these genomes (75%) harbored only putative additional genes and genes with no predicted function. These additional genes were identified by BAGEL4 as encoding modification proteins such as GlyS, a glycosyltransferase; regulatory proteins such as LanK, a sensor histidine kinase, and LanR, a transcriptional regulator; immunity/transport proteins such as ABC transporter and transport/leader cleavage proteins as LanT. BAGEL4 also predicted putative coding regions showing similarity to the Ref90 clusters, including kinases and acetyltransferases, in addition to genes with no predicted function (Figure 1). The genes required for lasso peptide production were identified in 27 rumen bacterial genomes using BAGEL4, however, only incomplete gene clusters were detected using BAGEL4 as no *lasB* gene was identified within these genomes using this computational tool (Table 1). Predictions of BGCs using antiSMASH 5 indicated gene clusters likely to encode lasso peptides in 33 ruminal bacterial genomes with all harboring at least *lasA*, *lasB*, or *lasC*. In 27 (82%) of these genomes, the gene *lasD* was also detected. These *in silico* screening approaches combined identified complete lasso peptide gene clusters in 11 rumen bacterial genomes, of which three were harbored by members of the genera *Bacillus*, *Butyrivibrio* and *Lachnospira*, and two in strains of the genus *Ruminococcus* (Table 1). These results show a higher prevalence and diversity of lasso peptides gene clusters within the *Firmicutes* phylum, mainly among members of the *Lachnospiraceae* family (Figure 1).

### Identification of Lasso Peptide Precursors in the Genomic Regions Surrounding the Biosynthetic Gene Clusters

BAGEL4 and/or antiSMASH 5 were effective to predict coding sequences homologous to the enzymes of the lasso peptide maturation pathway (LasB and LasC). However, the prediction of precursor sequences (LasA) was limited using these computational tools due to the inherent features of these peptides, such as their short length and sequence variability. We therefore investigated the genomic context of the gene clusters aiming to identify potential coding sequences containing the expected features of a lasso peptide precursor. This pattern-based search for novel lasso peptide precursors increased the number of rumen bacterial genomes harboring complete gene clusters to 33, including *Acetitomaculum ruminis* DSM 5522, *Eubacterium cellulosolvens* LD2006, two species of *Clostridium*, two species of *Lachnospira*, three strains of *Lachnospiraceae bacterium* and 13 strains of *Butyrivibrio*.

The genomes of *Butyrivibrio* sp. NC3005, *Butyrivibrio* sp. YAB3001, *Lachnospira multipara* LB2003, *Lachnospira pectinoschiza* M83, and *Lachnospiraceae bacterium* YSD2013 showed more than one sequence with homology to the *lasA* gene and careful examination of all 33 rumen bacterial genomes also indicated distinct sequences in the putative biosynthetic clusters that were likely to encode a lasso peptide precursor in *Butyrivibrio* sp. AB2020. Additionally, different *lasA* sequences were predicted in the genome of *Ruminococcus flavefaciens* 19208 by BAGEL4 and antiSMASH 5. The putative LasA proteins predicted for all the rumen bacterial genomes harboring complete lasso peptides gene cluster are reported in Supplementary Table 4.

**FIGURE 2:**
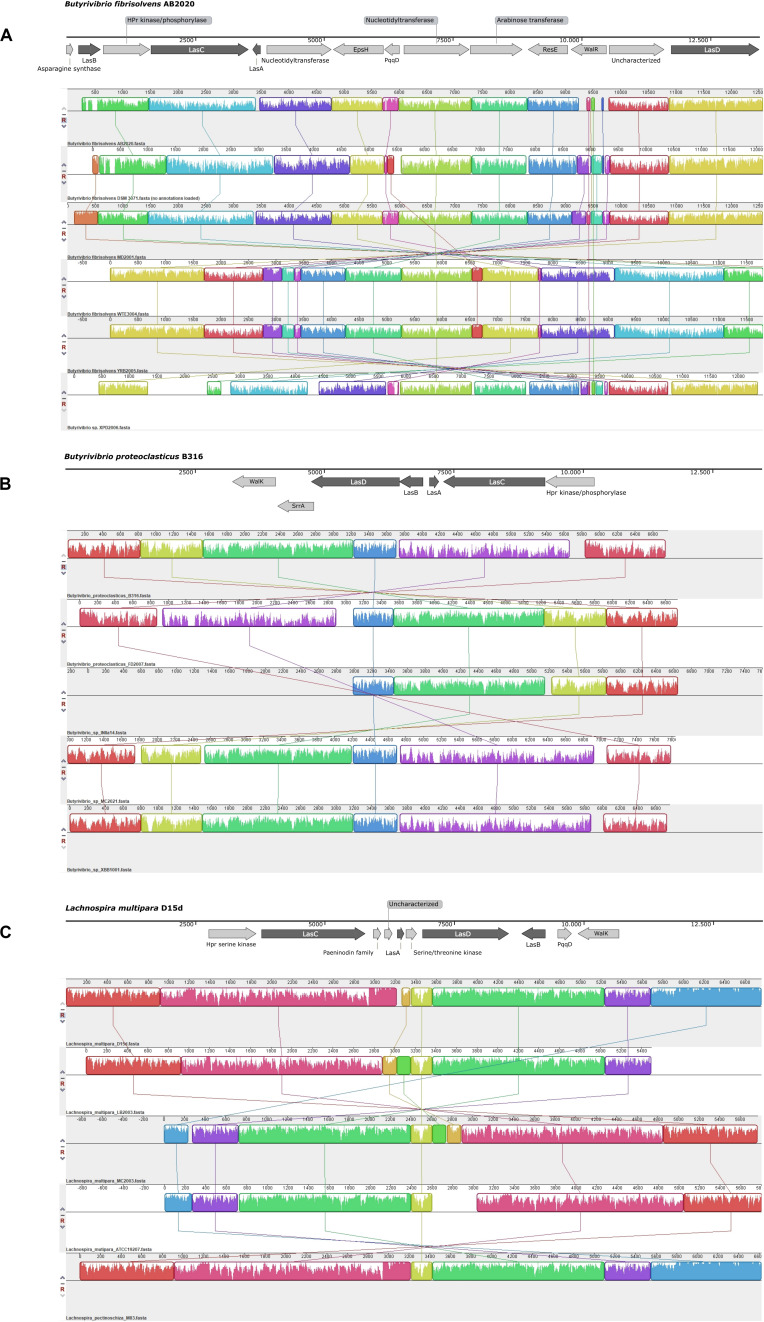
Schematic representation of the putative lasso peptide gene clusters and their conserved genomic context in strains of *Butyrivibrio* [Panels **(A)** and **(B)**] and *Lachnospira* [Panel **(C)**]. Two groups of putative lasso peptide gene clusters are observed in *Butyrivibrio*: Panel **(A)** shows the larger biosynthetic cluster, identified in *Butyrivibrio fibrisolvens*, while Panel **(B)** shows the genetic organization of the conserved lasso peptide clusters in the genomes of *Butyrivibrio proteoclasticus* and unclassified *Butyrivibrio* strains. When the biosynthetic cluster was identified in the reverse DNA strand, the alignment blocks are shown in the opposite direction. The genomic regions containing the biosynthetic gene clusters were aligned using Mauve version 20150226 and the representation of the genomic context was generated using SnapGene 5.0.8.

Expanding the genomic context analysis beyond the identification of *lasA* indicated that these biosynthetic clusters have a conserved pattern of genetic organization among groups of phylogenetically related organisms (Figure 2). Alignment of the lasso peptide gene clusters found in *Butyrivibrio fibrisolvens* indicated that these clusters contain additional genes such as nucleotidyltransferase, HPr kinase/phosphorylase, glycosyltransferase family 2 and EpsH, the sensor histidine kinase ResE and the transcriptional regulatory protein WalR. Other strains of *Butyrivibrio* sp. and *Butyrivibrio proteoclasticus* showed clusters containing the sensor protein kinase WalK, the transcriptional regulator SrrA, and the HPr kinase/phosphorylase, in addition to the essential biosynthetic genes. The lasso peptide gene cluster in strains of *Lachnospira* shared the serine kinase of the HPr protein and the sensor histidine kinase WalK, besides the putative genes encoding LasA, LasB, LasC, and LasD proteins.

Among the species of ruminal bacteria, the HPr kinase/phosphorylase, coenzyme PQQ synthesis protein D (PqqD), and an uncharacterized nucleotidyltransferase were found in approximately 58%, 46% and 30% of the genomes analyzed in this study, respectively (Supplementary Figure 2). Moreover, different proteins showing similar functional annotations were found within the lasso peptide gene clusters among these bacterial genomes, including the sensory proteins ResE and WalK, serine kinases, transcriptional regulators (WalR and SrrA) and glycosyltransferases (EpsH) (Supplementary Figure 2).

### Sequence Conservation Analysis of the Putative Lasso Peptide Proteins LasA, LasB, and LasC

Analysis of conserved elements in the lasso peptide biosynthesis machinery included LasA-like precursor peptides and proteins homologous to the lasso peptide maturation enzymes (LasB, LasC, and LasD). No conserved motifs were identified in the putative LasA amino acid sequences predicted in the genomes of ruminal bacteria analyzed in this study, indicating that these residues are hypervariable and therefore, subjected to neutral drift. However, genome mining enabled the identification of conserved motifs in homologs of the lasso peptide maturation enzymes LasB, LasC, and LasD, with variable frequency (Table 2). Two conserved motifs were identified in the LasB homologs, including a transglut_core3 motif of the transglutaminase-like superfamily, which is involved in the formation of lasso peptide amide crosslink and was found in 97% of the analyzed LasB proteins. Also, a MdlB motif of an ATPase and permease component of the ABC-type multidrug transport system was detected in the LasB homolog found in the genomes of *Ruminococcus albus* 8 and *Ruminococcus flavefaciens* ATCC 19208. In these genomes, the *lasB* gene appears to be fused with another gene encoding a transporter protein. The MdlB motif was also found in 87% of the LasD homologs predicted in the genomes of ruminal bacteria analyzed in this study, together with five other domains of different ABC-type transporters occurring at much lower abundances. Six motifs were found in the LasC homologs, most of which belong to a conserved protein domain family (asn_synth_AEB superfamily) of the glutamine-hydrolyzing asparagine synthases.

**TABLE 2:** Conserved motifs in lasso peptide maturation enzymes (LasB, LasC, and LasD) predicted in the genomes of ruminal bacteria.

Protein	Conserved motifs	Accession	Frequency	Coverage (%)
LasB	Transglut_core3	pfam13471	97%	45–100
	MdlB	COG1132	6%	100
LasC	AANH_like superfamily	cl00292	6%	100
	asn_synth_AEB superfamily	cl36928	74%	41–100
	Asn_Synthase_B_C	cd01991	18%	100
	AsnB	cd00712	6%	100
	AsnB superfamily	cl33852	6%	57–60
	Gn_AT_II superfamily	cl00319	15%	73–100
LasD	ABC_ATPase superfamily	cl25403	10%	98–100
	GlnQ	COG1126	3%	100
	HisM	COG0765	3%	100
	MdlB	COG1132	87%	89–100
	SunT superfamily	cl34455	3%	33
	UgpA	COG1175	3%	91

**FIGURE 3:**
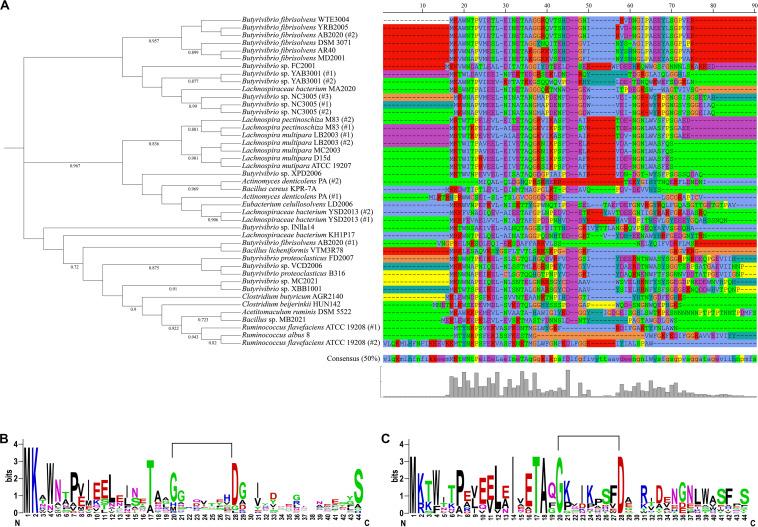
Phylogenetic and sequence conservation analysis of LasA-like precursors predicted in the genomes of ruminal bacteria. **(A)** Phylogenetic tree of the LasA-like peptides showing the amino acid sequences of each putative lasso peptide precursor. Sequences were aligned using muscle 3.8.31 and the phylogenetic tree was reconstructed using FastTree 2.1 (Maximum Likelihood, 1000 replicates). Annotation was performed using iTOL. Only bootstrap values greater than 70% are shown at the nodal branches. **(B)** and **(C)** show web logos of the putative LasA amino acid sequences predicted in genomes of the genera *Butyrivibrio* (*n* = 20) and *Lachnospira* (*n* = 7), respectively. The lines connecting the conserved glycine (G) and aspartate (D) residues in the lasso peptides precursor indicate the predicted location of the characteristic N-terminal macrolactam ring of the mature peptide.

Phylogenetic analysis of the predicted LasA precursor peptides grouped the sequences in three phylogroups each consisting of a different number of nodes (Figure 3A). The precursor peptides predicted in the genomes of most *Butyrivibrio* strains and all members of the genus *Lachnospira* analyzed in this study could be separated into three main distinct coherent clades. Sequences of lasso peptide precursors in the genomes of the genus *Ruminococcus* also generated a smaller and consistent clade within these phylogroups, while other sequences were spread throughout the phylogenetic tree. Although the overall amino acid sequence conservation was relatively low, residues located at positions 20–45 and 60–70 in the putative precursor peptides appeared with higher frequencies compared to other regions of the peptide (Figure 3A). As shown for the genomes of *Butyrivibrio* and *Lachnospira*, the 20–45 region contains a moderately to highly conserved glycine (G) residue spaced 8–9 positions away from an aspartate (D) residue, which are expected to form an isopeptide bond that installs the macrolactam into the N-terminus of the core peptide (Figures 3B,C). The glycine residue is also preceded at position 17 by a highly conserved threonine (T) residue that is often reported as an invariant residue present at the end of the leader peptides of lasso precursors (Knappe et al., 2009; Jia Pan et al., 2012; Hegemann et al., 2013a, 2014). Therefore, this pattern of conserved elements in the precursor sequences matches the chemical/structural properties that have been described for known lasso peptides, confirming their essential role in lasso peptide function and thereby ensuring the reliability of our outputs.

**FIGURE 4:**
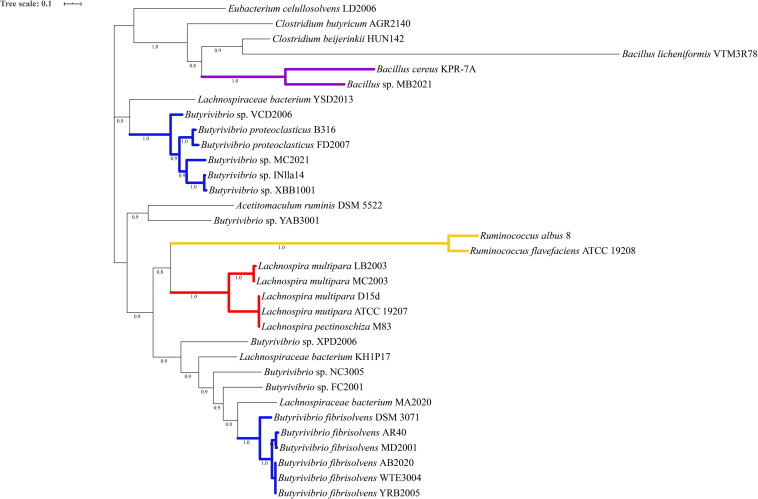
Phylogenetic tree showing putative LasB proteins predicted in the genomes of ruminal bacteria. Colored clades represent members of particular genera or species sharing a single common ancestor. Protein sequences were predicted by antiSMASH 5 and aligned using muscle 3.8.31. The phylogenetic tree was reconstructed using FastTree 2.1 (Maximum Likelihood, 1000 replicates). Tree annotation was performed using iTOL. Only bootstrap values greater than 70% are shown at the nodal branches.

**FIGURE 5:**
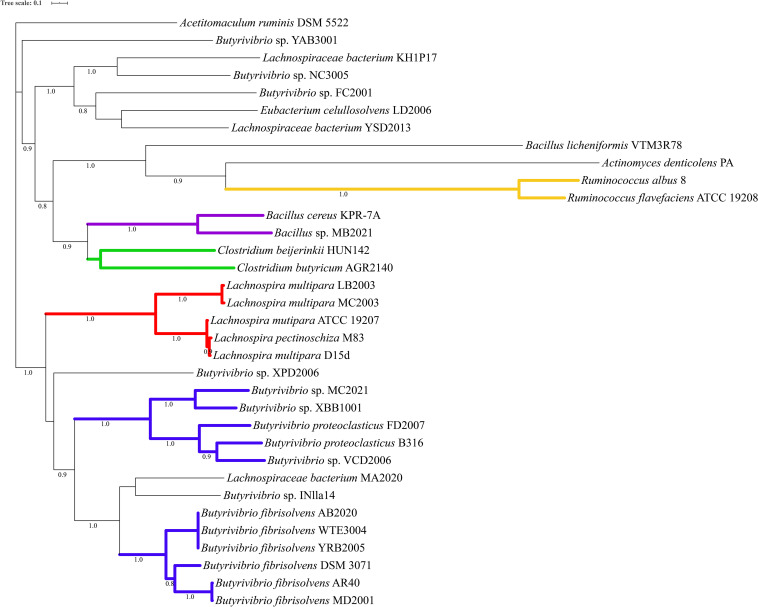
Phylogenetic tree showing putative LasC proteins predicted in the genomes of ruminal bacteria. Colored clades represent members of particular genera or species sharing a single common ancestor. Protein sequences were predicted by antiSMASH 5 and aligned using muscle 3.8.31. The phylogenetic tree was reconstructed using FastTree 2.1 (Maximum Likelihood, 1000 replicates). Tree annotation was performed using iTOL. Only bootstrap values greater than 70% are shown at the nodal branches.

Phylogenetic analysis of the proteins predicted as homologous of the lasso peptide maturation enzymes demonstrated that amino acid sequences of the LasB and LasC proteins belonging to the genera *Butyrivibrio*, *Lachnospira*, and *Ruminococcus* split into distinct phylogroups within the genomes of ruminal bacteria (Figures 4, 5). Also, sequences of the associated maturation enzymes appeared to be more conserved among the strains of *Butyrivibrio fibrisolvens* and *Lachnospira multipara*. The larger distancing of the LasB sequences observed in the clade containing members of the genus *Ruminococcus* is likely because these protein sequences are fused to a transporter protein.

### Prediction of Putative Lasso Peptide Precursors in the Genomes of Ruminal Bacteria

Combining our sequence conservation analysis of the putative lasso peptide precursors (LasA) with the requirement of specific residues distributed at particular positions in the amino acid sequences of the mature lasso peptides allowed the identification of novel sequences that were likely to be precursor hits in the genomes of ruminal bacteria. In total, thirty-five LasA-like precursors were predicted in 29 genomes of ruminal bacteria investigated in the current study (Table 3). In *Butyrivibrio* sp. NC3005, *Butyrivibrio* sp. YAB3001, *Lachnospira multipara* LB2003, *Lachnospira pectinoschiza* M83, *Lachnospiraceae bacterium* YSD2013 and *Ruminococcus flavefaciens* ATCC 19208, at least two distinct LasA precursors were predicted in each genome. The predicted core peptide sequences indicated that the N-terminal isopeptide-bonded ring is likely to occur between a glycine residue (G) at position + 1 of the core and an aspartate residue (D) positioned 8–9 aa from the beginning of the core. Also, an invariant threonine residue (T) that has been indicated as a recognition element for the lasso peptide maturation enzymes was found at the end of the predicted leader peptide (position -3 from the start of the core). Our analysis also revealed that most of the identified LasA candidates present a highly conserved serine (S) residue in their C-terminal region. The length of the putative core peptide sequences varied from 21 to 42 amino acid residues, according to the sizes of their respective gene coding sequences (Table 3).

**TABLE 3:** Amino acid sequences of putative lasso peptide precursors predicted in the genomes of ruminal bacteria.

Genome	Predicted leader peptide	Predicted core peptide	Core lenght
*Bacillus cereus* KPR-7A	MKKDWTIPTLEVLDINM**T**MA	**G**PGLKTP**D**AVQPDVDEVVHY**S**	21
*Butyrivibrio fibrisolvens* AB2020	MKAWNTPVIETLEINE**T**AA	**G**GRQVTSH**D**GNIRVDNGIPAEEYL**S**GPVER	29
*Butyrivibrio fibrisolvens* AR40	MKVWNAPVMESLEINE**T**AK	**G**GKKVSEH**D**GFINYSHNGLPAEEYA**S**GPVAK	31
*Butyrivibrio fibrisolvens* DSM 3071	MKAWNTPVMESLEINE**T**AG	**G**YNQITEH**D**GVINYSAGIPAEEYA**S**GPLPR	30
*Butyrivibrio fibrisolvens* MD2001	MKVWNAPVMESLEINE**T**AK	**G**GKKVSEH**D**GFINYSHNGLPAEEYA**S**GPVAK	31
*Butyrivibrio fibrisolvens* WTE3004	MKAWNTPVIETLEINE**T**AA	**G**GRQVTSH**D**GNIRVDNGIPAEEYL**S**GPVER	30
*Butyrivibrio fibrisolvens* YRB2005	MKAWNTPVIETLEINE**T**AA	**G**GRQVTSH**D**GNIRVDNGIPAEEYL**S**GPVER	30
*Butyrivibrio proteoclasticus* B316	MKNWNAPEIKELCLSG**T**QQH	**G**TPNPYV**D**GKIYDAQRNENWFTF**S**GNNVDDTATPGEVIIGNP	42
*Butyrivibrio proteoclasticus* FD2007	MKNWNTPEIKELSLSG**T**QLH	**G**QDVRFV**D**GSIYDEERNTNWASY**S**GGRPNRDEQPGEVIIH	40
*Butyrivibrio* sp. FC2001	MKKVWNDATLEALDITA**T**AG	**G**IYDTEEL**D**SEKWFDEEHKQWWG**S**FGNNNLSKAKKED	37
*Butyrivibrio* sp. INlla14	MKTWNSAEIVELALNQ**T**AG	**G**SFDITTH**D**GKIVYTTLANGRQVP**S**EQYAYVSGEQHA	37
*Butyrivibrio* sp. MC2021	MKNWNAPEIQELNLSN**T**EL	**G**NRRSGYC**D**AAVWSVELHKNFYSY**S**GEGDPNEDEWNVHPQH	41
*Butyrivibrio* sp. NC3005 (#1, #2)	MKWNAPVMEELNINA**T**AN	**G**MAPDENF**D**GDWVEINGKWYRPGNG**S**VSGGEIAQ	34
*Butyrivibrio* sp. NC3005 (#3)	MKWNAPVMEELNINA**T**AN	**G**MAPDENF**D**GDWVEINGRWYRPGNG**S**ISGSETAH	34
*Butyrivibrio* sp. VCD2006	MKKWNAPNIQELNLSS**T**MLR	**G**RNPKYV**D**GYVYDAERDTNWASY**S**GGTSDPSATGAEVIINNP	42
*Butyrivibrio* sp. XBB1001	MKTWTSPEIKELNISN**T**AL	**G**NARSGYC**D**AAVYSIELHRNFYSF**S**GEGEKNQDDWHVTPQNP	42
*Butyrivibrio* sp. XPD2006	MKTWVNPEVVELEISA**T**AQ	**G**DGPIAIP**D**AIRVDNDGTWYSFPSG**SS**QDAQ	31
*Butyrivibrio* sp. YAB3001 (#1)	MKTWLDAVIEEINFEK**T**ED	**G**RSEKLDN**D**RQYTDGRGLAIQLGGHL**S**	27
*Butyrivibrio* sp. YAB3001 (#2)	MKAWNTPVIEEVKFTA**T**KE	**G**SQQWQVP**D**KHYLDETDNQWKWEF**S**DGKLN	30
*Clostridium beijerinkii* HUN142	MNETKLKWSEPEVMDLSVKD**T**QL	**G**GHYSSSP**D**GAPWQDSNGNWQEPHGK**S**	18
*Eubacterium celullosolvens* LD2006	MKKWVNPEFKVLNISK**T**TY	**G**PWNQTIP**D**SELTAEFDEFGNVKGYRQLFGQASGTTGETGTPAV	44
*Lachnospira multipara* D15d	MKTWITPRVEELEIVE**T**AQ	**G**KSIKPSF**D**AFRIDENGNLWASFE**S**	25
*Lachnospira multipara* LB2003 (#1)	MRTWTKPEVEVLAIAE**T**AQ	**G**KVIKASF**D**AIRTDVNGNLWVSFP**S**GAEE	28
*Lachnospira multipara* LB2003 (#2)	MKTWITPAVEELEIVE**T**AQ	**G**KNIKPSF**D**ELRVDANGNLWASFQ**S**	25
*Lachnospira multipara* MC2003	MKTWITPAVEELEIVE**T**AQ	**G**KNIKPSF**D**ELRVDANGNLWASFQ**S**	25
*Lachnospira multipara* ATCC 19207	MKTWITPRVEELEIVE**T**AQ	**G**KSIKPSF**D**AFRIDENGNLWASFE**S**	25
*Lachnospira pectinoschiza* M83 (#1)	MKTWTKPELEVLAIEE**T**AQ	**G**KVIKPSF**D**SVRTDENGNLWASFP**S**GAEE	29
*Lachnospira pectinoschiza* M83 (#2)	MRTWTTPELEVLEITE**T**AQ	**G**KVIKASF**D**AIRTDENGNLWVSFP**S**GAED	29
*Lachnospiraceae bacterium* KH1P17	MRKWNTPELQELAINA**T**AG	**G**PQDNHTE**D**GKTVYDHEENAWYHRFGEDGNYTRN	34
*Lachnospiraceae bacterium* MA2020	MKSWNTPAIEELNINE**T**AG	**G**GQKTMNW**D**GEWITPDEGK**S**WWAGTVIV**S**G	30
*Lachnospiraceae bacterium* YSD2013 (#1)	MKKFEVAELVVLNIAD**T**AF	**G**PDDPNHVD**D**YKHAVEDPITHEVLGYEEEYGQAH**SS**NQN	39
*Lachnospiraceae bacterium* YSD2013 (#2)	MKKFVNADIQEVAIEE**T**AF	**G**PLNPEDV**D**ETKYAVTDEEGNIIGYRAKFGKADA**S**RQ	37
*Ruminococcus albus* 8	MTYNKPSFEKISSFKES**T**M	**G**VWFGKFK**D**IFGGKAVVEIVIYY	23
*Ruminococcus flavefaciens* ATCC 19208 (#1)	MTYNKPSVEKVASFKDN**T**M	**G**LWYGKFR**D**IFGAKTYFNLAWN	22
*Ruminococcus flavefaciens* ATCC 19208 (#2)	MTYNKPSFEKVASFKNK**T**M	**G**LWFGNFK**D**LFGGKIYIAL**S**PAW	23

*The precursor peptides are composed by the leader peptide + core peptide. Letters in bold represent conserved amino acid residues in the leader and core peptides involved in the maturation or post-translational modification of the lasso peptides. Numbers inside brackets were added when more than one LasA sequence were found in the same genome.*

### Expression Analysis of Putative Lasso Peptides Biosynthetic Genes in Ruminal Metatranscriptome Datasets

Expression of the putative lasso peptide precursors and associated maturation enzymes were generally low in the ruminal metatranscriptome datasets, varying from 0.01 to 4.26 reads per kilobase per million of mapped reads (RPKM). Nonetheles, the expression levels differed between the genes *lasA*, *lasB*, and *lasC* and the number of mapped reads also varied among the datasets of beef cattle, dairy cattle, and sheep examined in the current study. Overall, only reads corresponding to the genes *lasA, lasB*, and *lasC* of *Ruminococcus albus* 8 were represented simultaneously in at least two datasets under study (SRR873462 and SRR873454).

**FIGURE 6:**
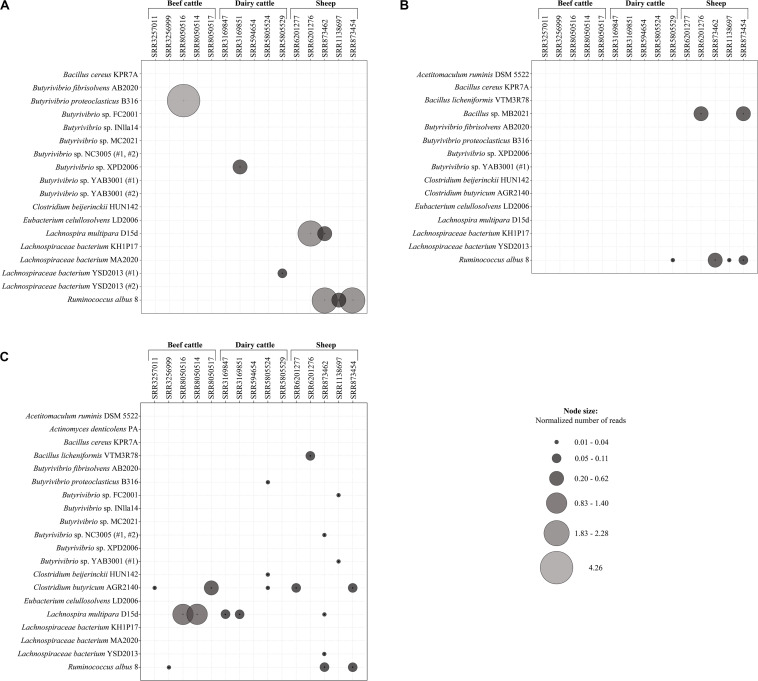
Expression of putative *lasA* **(A)**, *lasB* **(B)**, and *lasC* **(C)** genes in different rumen metatranscriptomes. Node sizes represent the relative abundance of reads (expressed as RPKM) mapped to the corresponding bacterial genome in each dataset. The ruminal metatranscriptomes of dairy cattle, beef cattle, and sheep were obtained from the sequence read archive (SRA) in NCBI. The analyses were performed using Bowtie and the alignment was visualized in the software Tablet 1.19.09.03.

The gene *lasC* showed the highest number of reads mapping to a putative lasso peptide biosynthetic gene within the active ruminal microbial community, being detected in 13 out of the 15 datasets (Figure 6C). Only reads mapping to the *lasC* gene of *Lachnospira multipara* D15d, *Clostridium butyricum* AGR2140, and *Ruminococcus albus* 8 were represented in the rumen of beef cattle, while for dairy cattle datasets, the mapped reads represented the *lasC* gene found in *Butyrivibrio proteoclasticus* B316, *Lachnospira multipara* D15d, and two species of *Clostridium*. The *lasC* gene also appears to be more broadly expressed in the ruminal metatranscriptomes of sheep, as reads of the genes that were found in eight bacterial genomes mapped to at least one out of the five datasets analyzed. Moreover, the expression of the *lasC* gene found in *Clostridium butyricum* ARG2140 and *Lachnospira multipara* D15d occurred across all the dataset groups (beef cattle, dairy cattle, and sheep) evaluated in the current study.

Reads mapping to the predicted *lasA* genes showed higher average RPKM values among the metatranscriptomes evaluated in the current study (Figure 6A). The highest RPKM value (4.26) was observed for reads mapping to the *lasA* sequences of *Butyrivibrio proteoclasticus* B316 in a beef cattle metatranscriptome. Only *lasA* reads from *Butyrivibrio* sp. XPD2006 and *Lachnospiraceae bacterium* YSD2013 (#1) were aligned to the dairy cattle metatranscriptomes. In the active ruminal microbial community of sheep, reads representing the *lasA* sequences of *Lachnospira multipara* D15d and *Ruminococcus albus* 8 mapped to two and three of the datasets under study, respectively.

Among the essential genes of the lasso peptide biosynthesis machinery, the genes corresponding to the maturation enzyme *lasB* were the least expressed across all the ruminal datasets examined in the current study. Only the reads corresponding to the *lasB* gene of *Bacillus* sp. MB2021 and *Ruminococcus albus* 8 could be mapped to a ruminal metatranscriptome, with the majority of the sequences aligned in the dairy cattle and sheep datasets (Figure 6B).

## Discussion

Lasso peptides represent a group of ribosomally synthesized and post-translationally modified peptides (RiPPs) with unique topology and diverse biological activities. The discovery of new lasso peptides may be hindered by the great sequence variation of these molecules. Previously, we applied genome mining approaches to explore the ruminal environment as a potential source of bacteriocins (Azevedo et al., 2015), and non-ribosomal peptides and polyketides (Moreira et al., 2020). Here, we performed an extensive *in silico* screen in publically available genomes of ruminal bacteria in an attempt to determine the prevalence and diversity of lasso peptides among major species of bacteria from the rumen. Additionally, we sought out to predict potentially novel lasso peptides in the genomes of different members of the rumen microbiome, thus guiding the future identification and characterization of these antimicrobial peptides in ruminal bacteria through *in vitro* studies.

The genetic screening using antiSMASH 5 and BAGEL4 revealed putative clusters for lasso peptides biosynthesis in 34 ruminal bacterial genomes, representing 8% of the total analyzed. From these, only 11 genomes presented clusters harboring all the essential genes of the biosynthesis machinery of lasso peptides, while in the other genomes the biosynthetic clusters were incomplete. However, after manually curating the genomic context of the incomplete gene clusters, thirty-three rumen bacterial genomes were found harboring complete lasso peptide clusters, confirming the limitations of using homology search tools to identify these biosynthetic genes through automated mining of complete and draft genomes. It should be emphasized, however, that both antiSMASH 5 and BAGEL4 are valuable tools for analyzing and identifying biosynthetic gene clusters in microbial genomes due to their intrinsic processivity, which allows fast screening of multiple datasets of genomic sequences. Nonetheless, computational tools may be limited by inherent constraints of their search algorithms, representativeness of the databases, and by singularities of the lasso peptide gene clusters, such as their small sizes and hypervariability of the precursor gene sequences (Tietz et al., 2017). Therefore, the examination of the genomic context flanking the essential genes may be a useful approach to refine pattern-based genome mining.

Lasso peptide production has been mainly associated with members of the phyla *Actinobacteria* and *Proteobacteria* and the best-studied molecules of this family are produced by representative species within these phyla (Weber et al., 1991; Salomon and Farías, 1992; Helynck et al., 1993; Potterat et al., 1994; Tsunakawa et al., 1995; Kimura et al., 1997; Knappe et al., 2008; Hegemann et al., 2013a). Moreover, genome-guided studies focused on natural product discovery often point to these bacterial phyla as promising sources of lasso peptides (Severinov et al., 2007; Hegemann et al., 2013b; Maksimov and Link, 2014). However, our results indicate that genera of the phylum *Firmicutes* are probably the main producers of lasso peptides in the rumen environment, revealing these taxa as potential sources of new lasso peptides. These results are in agreement with a genome-guided exploration of the GenBank database, which highlighted the phyla *Firmicutes*, *Cyanobacteria*, *Euryarchaeota*, and *Bacteroidetes* as underexplored sources of lasso peptides (Tietz et al., 2017). The higher prevalence of lasso peptide gene clusters within the phylum *Firmicutes* reported in this study must take into account the abundance of sequenced genomes compared to other phyla of ruminal bacteria. Nonetheless, the presence of complete biosynthetic clusters observed in multiple strains of the phylum *Firmicutes* may increase the interest in exploring members of these taxa to accelerate the discovery of new bioactive molecules through *in silico* analyses and *in vitro* experimentation.

Analyses of the genomic context allowed the identification of additional co-occurring genes frequently found associated with the lasso peptide biosynthetic gene clusters, such as kinases, glycosyltransferases, and nucleotidyltransferases (Duquesne et al., 2007b; Tietz et al., 2017; Zyubko et al., 2019). These co-occurring genes provide additional evidence of the probable functionality of the gene clusters identified in the rumen bacterial genomes and their association with the essential biosynthesis genes appears to be characteristic among species of the phylum *Firmicutes* (Zhu et al., 2016c). Some of the additional genes flanking the biosynthetic clusters encode tailoring enzymes that introduce chemical modifications to the lasso peptides, including covalent modifications in their C-terminal region, such as phosphorylation (Zhu et al., 2016c), methylation (Gavrish et al., 2014) or acetylation (Zong et al., 2018). For example, HPr kinases found in *Paenibacillus dendritiformis* C454 are capable of modifying a C−terminal Ser in the lasso peptide precursor by adding one or more phosphate groups (Zhu et al., 2016b,c). In *Bacillus pseudomycoides* DSM 12442, producer of pseudomycoidin, the presence of the PsmN, a nucleotidyltransferase, was associated with the glycosylation of the C-terminal region (Zyubko et al., 2019). The advantages that these chemical modifications offer to the lasso peptides remains unclear, but they may affect peptide stability (Zyubko et al., 2019), regulate critical processes, such as signaling pathways (Zhu et al., 2016b) or influence the functioning of self-immunity systems in the producer cells (Zhu et al., 2016c; Zong et al., 2018).

A gene encoding a protein belonging to the PqqD enzyme superfamily frequently co-occurred with the lasso peptide biosynthesis genes in species of the genera *Butyrivibrio* and *Lachnospira*. It has been previously reported that the maturation enzyme B can be split between two separate open reading frames (proteins B1 and B2), and this genetic configuration seems a common feature in the lasso peptide gene clusters found in Gram-positive bacteria (Maksimov and Link, 2014; Cheung et al., 2016). Protein B1 is annotated as a homolog of the PqqD enzyme superfamily (Zhu et al., 2016a; Sumida et al., 2019), and acts as a RiPP recognition element that binds the leader peptide and delivers the precursor for processing, while protein B2 is homologous to the transglutaminases and is responsible for the processing of the precursor peptide.

The predicted products of the putative biosynthetic genes detected by antiSMASH 5 and BAGEL4 were subjected to motif analysis and results demonstrated that the LasB homologs found in the rumen bacterial genomes harbor conserved motifs of the transglutaminases family, while the predicted LasC homologs contain consensus motifs of the asparagine synthetase family. These analyses are in agreement with the genome mining data and provide evidence for the presence of conserved catalytic domains on the enzymes of the lasso peptide maturation pathway. The transglutaminases belong to a large family of cysteine proteases capable of catalyzing the formation of amide crosslinks (Makarova et al., 1999). These enzymes contain the Cys-His-Asp catalytic triad in their active site, which is conserved among the McjB and McjB-like proteins from various bacteria (Duquesne et al., 2007a). Evidence of this catalytic triad was also found on the LasB homologs predicted in the genomes of rumen bacteria investigated in the current study, being particularly conserved in species of *Butyrivibrio* (Supplementary Figure 3). In the species of *Ruminococcus*, the LasB homologs contained a fused ABC-transporter domain, a feature that has been previously described for other maturation enzymes of this group (Maksimov and Link, 2014; Tietz et al., 2017).

The phylogenetic analysis of the putative proteins encoded by the *lasA*, *lasB*, and *lasC* genes revealed monophyletic branches composed of strains of the same genera/species. Additionally, the genomic context analysis indicated that the lasso peptide clusters of phylogenetic-related bacteria show the same patterns of genetic organization and considerable conservation of the biosynthetic genes, suggesting that the lasso peptide biosynthetic genes can be inherited vertically, as proposed previously (Tietz et al., 2017).

Determination of putative *lasA* genes can be performed screening short ORFs flanking the biosynthetic genes and considering the requirement of specific amino acids at particular positions of the peptide precursor for the macrolactam ring formation (Knappe et al., 2008; Hegemann et al., 2013a). Besides, our pattern-based genome mining considered the high conservation of the amino acids involved in the amide bond and the genomic context conservation of the lasso peptide clusters. It has been demonstrated that despite the high sequence variation within the structural gene *lasA*, the amino acids that are involved in the formation of the macrolactam ring are conserved across different species of bacteria (Hegemann et al., 2013a; Zhu et al., 2016c). The presence of a conserved threonine residue near the cleavage site of the leader peptide may be required for the recognition and binding of the protease, which seems a crucial event for the effective processing of some lasso peptides precursors (Knappe et al., 2009; Jia Pan et al., 2012; Hegemann et al., 2013a, 2014). Phylogenetic analysis of the LasA sequences found in ruminal bacterial genomes confirmed the conservation of these nearly invariant amino acid residues providing further evidence that they may be related to the lasso peptide function even though some of the sequences were larger than expected. Moreover, phylogenetic analysis of known lasso peptides and the core peptide sequences described in the current study (Supplementary Figure 4) reinforces the novelty of the LasA-like sequences discovered in the genomes of ruminal bacteria, since the sequences diverged from a common ancestor when compared to the clades that grouped other lasso peptide families.

Some of the bacteria identified in the current study carrying a putative gene cluster for lasso peptide production are also potential producers of other antimicrobial compounds. For example, *Ruminococcus albus* 8 produces an antagonistic thermostable substance capable of inhibiting *Ruminococcus flavefaciens* (Odenyo et al., 1994). In addition, two *g*ene clusters of sactipeptides and one cluster of a class III bacteriocin were found *in Ruminococcus albus* 8 (Azevedo et al., 2015). Bacteriocin gene clusters were also identified in the genomes of *Butyrivibrio fibrisolvens* MD2001, *Butyrivibrio proteoclasticus* B316, *Butyrivibrio proteoclasticus* FD2007, and *Lachnospira multipara* MC2003 (Azevedo et al., 2015), while genes associated with the biosynthesis of non-ribosomal peptides (NRP) and polyketides (PK) were reported in the genomes of *Bacillus cereus* KPR-7A and *Clostridium beijerinckii* HUN142 (Moreira et al., 2020). Altogether, the presence of gene clusters encoding for distinct classes of antimicrobial compounds in ruminal bacteria suggest the importance of these molecules in the rumen ecosystem and highlight their potential applications in controlling undesirable bacteria.

The genes considered essential to lasso peptide biosynthesis showed a low level of expression in the ruminal metatranscriptome datasets and were more expressed within the microbial community of the sheep rumen, which is in agreement with our previous observations indicating that non-ribosomal peptide synthetases (NRPS) and polyketide synthases (PKS) were also more abundant in the rumen microbiota of sheep (Moreira et al., 2020). Additionally, the reads corresponding to the genes found in *Butyrivibrio* sp., *Lachnospira* sp., and *Ruminococcus* sp. were most represented in the sheep metatranscriptomes, also confirming previous observations showing a higher prevalence of NRPS and PKS within members of these ruminal taxa (Moreira et al., 2020). Taken together, these results demonstrate the potential production of antimicrobial compounds by species of bacteria colonizing the rumen of sheep, which deserves further investigation.

The metatranscriptome analysis also revealed differences in the expression level of the essential lasso peptide biosynthetic genes (*lasA*, *lasB*, and *lasC*). Reads mapping simultaneously to all three essential genes was only observed for *Ruminococcus albus* 8 in two datasets from sheep, indicating that in these microbial communities complete biosynthesis machinery could be generated for lasso peptide production. Overall, reads mapping to the putative LasA precursors were more abundant than reads mapping to the maturation enzymes LasB and LasC. These differences may be due to regulation at transcriptional level controlling the gene expression, differences in RNA stability, or other mechanisms that are known to affect the biosynthesis of antimicrobial peptides and other natural products (Hindre et al., 2004; Trmcic et al., 2011).

The genome mining of lasso peptide biosynthetic clusters in genomes of ruminal bacteria confirmed that *in silico* screening is a fast, effective, and less expensive approach for discovery of these antimicrobial peptides. However, future research should further develop and confirm these initial findings through experiments designed to validate *in vitro* the production of lasso peptides by ruminal bacteria or heterologous expression of the predicted lasso peptides and to demonstrate their biological activity. Nonetheless, these computational analyses allow to narrow down substantially the genera/species potentially producing lasso peptides in the rumen ecosystem, thus guiding future culture-based efforts. Besides, the peptides predicted in the genomes of ruminal bacteria can serve as scaffolds to develop derivatives with improved antimicrobial activity, stability to proteases and lower cytotoxicity (Pan et al., 2010; Soudy et al., 2012), through chemical synthesis or heterologous expression without the need of culturing the producer organism. Overall, our results reveal that ruminal bacteria harbor the genetic arsenal necessary for the production of several not yet described lasso peptides. The discovery of new peptides from ruminal bacteria reinforces the potential of the rumen microbiome as an important source of secondary metabolites that require future characterization.

## Data Availability Statement

The datasets presented in this study can be found in online repositories. The names of the repository/repositories and accession number(s) can be found in the article/Supplementary Material.

## Author Contributions

HM, FA, SH, and TM conceived the project. YS, KA, TL, FA, TM, and SM performed the data analysis and interpretation of results under the supervision of HM. YS, KA, and HM wrote the manuscript. All authors read and approved the final manuscript.

## Conflict of Interest

The authors declare that the research was conducted in the absence of any commercial or financial relationships that could be construed as a potential conflict of interest.

## Footnotes

1 http://www.rmgnetwork.org/hungate1000.html

2 http://www.ncbi.nlm.nih.gov/genome

3 http://genome.jgi.doe.gov

4 https://rdp.cme.msu.edu/

5 https://iTOL.embl.de/

6 https://weblogo.berkeley.edu/logo.cgi

7 http://www.nii.ac.in/~priyesh/lantipepDB/new_predictions/index.php#/~priyesh/lantipepDB/new_predictions/cyclizationPrediction.php

8 https://www.ncbi.nlm.nih.gov/sra

## References

[B1] Agrawal P. Khater S. Gupta M. Sain N. Mohanty D. (2017). RiPPMiner: a bioinformatics resource for deciphering chemical structures of RiPPs based on prediction of cleavage and cross-links. *Nucleic Acids Res.* 45 W80–W88. 10.1093/nar/gkx408 28499008 PMC5570163

[B2] Arnison P. G. Bibb M. J. Bierbaum G. Bowers A. A. Bugni T. S. Bulaj G. (2013). Ribosomally synthesized and post-translationally modified peptide natural products: overview and recommendations for a universal nomenclature. *Nat. Prod. Rep.* 30 108–160. 10.1039/c2np20085f 23165928 PMC3954855

[B3] Azevedo A. C. Bento C. B. P. Ruiz J. C. Queiroz M. V. Mantovani H. C. (2015). Distribution and genetic diversity of bacteriocin gene clusters in rumen microbial genomes. *Appl. Environ. Microbiol.* 81 7290–7304. 10.1128/AEM.01223-15 26253660 PMC4579445

[B4] Bachmann B. O. Van Lanen S. G. Baltz R. H. (2014). Microbial genome mining for accelerated natural products discovery: is a renaissance in the making? *J. Ind. Microbiol. Biotechnol.* 41 175–184. 10.1007/s10295-013-1389-9 24342967 PMC4070288

[B5] Bayro M. J. Mukhopadhyay J. Swapna G. V. T. Huang J. Y. Ma L.-C. Sineva E. (2003). Structure of antibacterial peptide microcin J25: a 21-Residue lariat protoknot. *J. Am. Chem. Soc.* 125 12382–12383. 10.1021/ja036677e 14531661

[B6] Blin K. Shaw S. Steinke K. Villebro R. Ziemert N. Lee S. Y. (2019). antiSMASH 5.0: updates to the secondary metabolite genome mining pipeline. *Nucleic Acids Res.* 47 W81–W87. 10.1093/nar/gkz310 31032519 PMC6602434

[B7] Bountra K. Hagelueken G. Choudhury H. G. Corradi V. El Omari K. Wagner A. (2017). Structural basis for antibacterial peptide self-immunity by the bacterial ABC transporter McjD. *Embo J.* 36 3062–3079. 10.15252/embj.201797278 28864543 PMC5641919

[B8] Cheung W. L. Chen M. Y. Maksimov M. O. Link A. J. (2016). Lasso peptide biosynthetic protein larb1 binds both leader and core peptide regions of the precursor protein LarA. *ACS Cent. Sci.* 2 702–709. 10.1021/acscentsci.6b00184 27800552 PMC5084080

[B9] Constantine K. L. Friedrichs M. S. Detlefsen D. Nishio M. Tsunakawa M. Furumai T. (1995). High-resolution solution structure of siamycin II: novel amphipathic character of a 21-residue peptide that inhibits HIV fusion. *J. Biomol. NMR* 5 271–286. 10.1007/BF00211754 7787424

[B10] Duquesne S. Destoumieux-Garzón D. Zirah S. Goulard C. Peduzzi J. Rebuffat S. (2007a). Two enzymes catalyze the maturation of a lasso peptide in *Escherichia coli*. *Chem. Biol.* 14 793–803. 10.1016/j.chembiol.2007.06.004 17656316

[B11] Duquesne S. Petit V. Peduzzi J. Rebuffat S. (2007b). Structural and functional diversity of microcins, gene-encoded antibacterial peptides from enterobacteria. *J. Mol. Microbiol. Biotechnol.* 13 200–209. 10.1159/000104748 17827970

[B12] Frechet D. Guitton J. D. Herman F. Faucher D. Helynck G. Monegier du Sorbier B. (1994). Solution structure of RP 71955, a new 21 amino acid tricyclic peptide active against HIV-1 virus. *Biochemistry* 33 42–50. 10.1021/bi00167a006 8286361

[B13] Gavrish E. Sit, Clarissa S. Cao S. Kandror O. Spoering A. (2014). Lassomycin, a ribosomally synthesized cyclic peptide, kills mycobacterium tuberculosis by targeting the ATP-Dependent Protease ClpC1P1P2. *Chem. Biol.* 21 509–518. 10.1016/j.chembiol.2014.01.014 24684906 PMC4060151

[B14] Hegemann J. D. Zimmermann M. Xie X. Marahiel M. A. (2013a). Caulosegnins I–III: a highly diverse group of lasso peptides derived from a single biosynthetic gene cluster. *J. Am. Chem. Soc.* 135 210–222. 10.1021/ja308173b 23214991

[B15] Hegemann J. D. Zimmermann M. Zhu S. Klug D. Marahiel M. A. (2013b). Lasso peptides from *proteobacteria*: genome mining employing heterologous expression and mass spectrometry. *Pep. Sci.* 100 527–542. 10.1002/bip.22326 23897438

[B16] Hegemann J. D. Zimmermann M. Zhu S. Steuber H. Harms K. Xie X. (2014). Xanthomonins I–III: a new class of lasso peptides with a seven-residue macrolactam ring. *Angew. Chem. Int. Ed.* 53 2230–2234. 10.1002/anie.201309267 24446383

[B17] Helynck G. Dubertret C. Mayaux J.-F. Leboul J. (1993). Isolation of RP 71955, a new anti-HIV-1 peptide secondary metabolite. *J. Antibiot.* 46 1756–1757. 10.7164/antibiotics.46.1756 8270499

[B18] Hindre T. Le Pennec J. P. Haras D. Dufour A. (2004). Regulation of lantibiotic lacticin 481 production at the transcriptional level by acid pH. *FEMS Microbiol. Lett.* 231 291–298. 10.1016/S0378-1097(04)00010-2 14987777

[B19] Jia Pan S. Rajniak J. Maksimov O. M. James Link A. (2012). The role of a conserved threonine residue in the leader peptide of lasso peptide precursors. *ChemComm.* 48 1880–1882. 10.1039/c2cc17211a 22222556

[B20] Katahira R. Yamasaki M. Matsuda Y. Yoshida M. (1996). MS-271, A novel inhibitor of calmodulin-activated myosin light chain kinase from Streptomyces sp.—II. Solution structure of MS-271: characteristic features of the ‘lasso’ structure. *Bioorg. Med. Chem.* 4 121–129. 10.1016/0968-0896(95)00176-X 8689232

[B21] Kimura K.-I. Kanou F. Takahashi H. Esumi Y. Uramoto M. Yoshihama M. (1997). Propeptin, a new inhibitor of prolyl endopeptidase produced by microbispora. I. Fermentation, isolation and biological properties. *J. Antibiot.* 50 373–378. 10.7164/antibiotics.50.373 9207905

[B22] Knappe T. A. Linne U. Robbel L. Marahiel M. A. (2009). Insights into the biosynthesis and stability of the lasso peptide capistruin. *Chem. Biol.* 16 1290–1298. 10.1016/j.chembiol.2009.11.009 20064439

[B23] Knappe T. A. Linne U. Xie X. Marahiel M. A. (2010). The glucagon receptor antagonist BI-32169 constitutes a new class of lasso peptides. *FEBS Lett.* 584 785–789. 10.1016/j.febslet.2009.12.046 20043911

[B24] Knappe T. A. Linne U. Zirah S. Rebuffat S. Xie X. Marahiel M. A. (2008). Isolation and structural characterization of capistruin, a lasso peptide predicted from the genome sequence of *Burkholderia thailandensis* E264. *J. Am. Chem. Soc.* 130 11446–11454. 10.1021/ja802966g 18671394

[B25] Knappe T. A. Manzenrieder F. Mas-Moruno C. Linne U. Sasse F. Kessler H. (2011). Introducing lasso peptides as molecular scaffolds for drug design: engineering of an integrin antagonist. *Angew. Chem. Int. Ed.* 50 8714–8717. 10.1002/anie.201102190 21812076

[B26] Kuznedelov K. Semenova E. Knappe T. A. Mukhamedyarov D. Srivastava A. Chatterjee S. (2011). The antibacterial threaded-lasso peptide capistruin inhibits bacterial RNA polymerase. *J. Mol. Biol.* 412 842–848. 10.1016/j.jmb.2011.02.060 21396375 PMC3143284

[B27] Langmead B. Salzberg S. L. (2012). Fast gapped-read alignment with Bowtie 2. *Nat. Methods* 9 357–359. 10.1038/nmeth.1923 22388286 PMC3322381

[B28] Larsen T. M. Boehlein S. K. Schuster S. M. Richards N. G. J. Thoden J. B. Holden H. M. (1999). Three-dimensional structure of *Escherichia coli* asparagine synthetase B: a short journey from substrate to product. *Biochemistry* 38 16146–16157. 10.1021/bi9915768 10587437

[B29] Leinonen R. Sugawara H. Shumway M. International Nucleotide Sequence Database (2011). The sequence read archive. *Nucleic Acids Res.* 39 D19–D21. 10.1093/nar/gkq1019 21062823 PMC3013647

[B30] Letunic I. Bork P. (2019). Interactive tree of life (iTOL) v4: recent updates and new developments. *Nucleic Acids Res.* 47 W256–W259. 10.1093/nar/gkz239 30931475 PMC6602468

[B31] Lu S. Wang J. Chitsaz F. Derbyshire M. K. Geer R. C. Gonzales N. R. (2020). CDD/SPARCLE: the conserved domain database in 2020. *Nucleic Acids Res.* 48 D265–D268. 10.1093/nar/gkz991 31777944 PMC6943070

[B32] Makarova K. S. Aravind L. Koonin E. V. (1999). A superfamily of archaeal, bacterial, and eukaryotic proteins homologous to animal transglutaminases. *Protein Sci.* 8 1714–1719. 10.1110/ps.8.8.1714 10452618 PMC2144420

[B33] Maksimov M. O. Link A. J. (2014). Prospecting genomes for lasso peptides. *J. Ind. Microbiol. Biotechnol.* 41 333–344. 10.1007/s10295-013-1357-4 24142336

[B34] Maksimov M. O. Pan S. J. Link A. J. (2012a). Lasso peptides: structure, function, biosynthesis, and engineering. *Nat. Prod. Rep.* 29 996–1006. 10.1039/c2np20070h 22833149

[B35] Maksimov M. O. Pelczer I. Link A. J. (2012b). Precursor-centric genome-mining approach for lasso peptide discovery. *Proc. Natl. Acad. Sci. U.S.A.* 109 15223–15228. 10.1073/pnas.1208978109 22949633 PMC3458324

[B36] Mantovani H. C. Hu H. Worobo R. W. Russell J. B. (2002). Bovicin HC5, a bacteriocin from *Streptococcus bovis* HC5. *Microbiology* 148(Pt 11), 3347–3352. 10.1099/00221287-148-11-3347 12427926

[B37] Milne I. Stephen G. Bayer M. Cock P. J. Pritchard L. Cardle L. (2012). Using Tablet for visual exploration of second-generation sequencing data. *Brief. Bioinform.* 14 193–202. 10.1093/bib/bbs012 22445902

[B38] Morais S. Mizrahi I. (2019). The road not taken: the rumen microbiome, functional groups, and community states. *Trends Microbiol.* 27 538–549. 10.1016/j.tim.2018.12.011 30679075

[B39] Moreira S. M. de Oliveira Mendes T. A. Santanta M. F. Huws S. A. Creevey C. J. Mantovani H. C. (2020). Genomic and gene expression evidence of nonribosomal peptide and polyketide production among ruminal bacteria: a potential role in niche colonization? *FEMS Microbiol. Ecol.* 96:fiz198. 10.1093/femsec/fiz198 31825517

[B40] Mortazavi A. Williams B. A. McCue K. Schaeffer L. Wold B. (2008). Mapping and quantifying mammalian transcriptomes by RNA-Seq. *Nat. Methods* 5 621–628. 10.1038/nmeth.1226 18516045 PMC13303166

[B41] Neumann A. P. Suen G. (2018). The phylogenomic diversity of herbivore-associated fibrobacter spp. Is Correlated to Lignocellulose-Degrading Potential. *mSphere* 3:e00593-18. 10.1128/mSphere.00593-18 30541780 PMC6291624

[B42] Newman D. J. Cragg G. M. (2016). Natural products as sources of new drugs from 1981 to 2014. *J. Nat. Prod.* 79 629–661. 10.1021/acs.jnatprod.5b01055 26852623

[B43] Odenyo A. A. Mackie R. I. Stahl D. A. White B. A. (1994). The use of 16S rRNA-targeted oligonucleotide probes to study competition between ruminal fibrolytic bacteria: development of probes for Ruminococcus species and evidence for bacteriocin production. *Appl. Environ. Microb.* 60 3688–3696. 10.1128/aem.60.10.3688-3696.1994 7527201 PMC201874

[B44] Oyama L. B. Girdwood S. E. Cookson A. R. Fernandez-Fuentes N. Prive F. Vallin H. E. (2017). The rumen microbiome: an underexplored resource for novel antimicrobial discovery. *NPJ Biofilms Microbiomes* 3 1–9. 10.1038/s41522-017-0042-1 29214045 PMC5711939

[B45] Oyama L. B. Olleik H. Teixeira A. C. N. Guidini M. M. Pickup J. A. Cookson A. R. (2019). In silico identification of novel peptides with antibacterial activity against multidrug resistant Staphylococcus aureus. *BioRxiv* Available online at: https://www.biorxiv.org/content/10.1101/577221v1 (accessed on 24 June 2020). 10.1038/s41522-022-00320-0 PMC9283466 35835775

[B46] Palevich N. Kelly W. J. Leahy S. C. Denman S. Altermann E. Rakonjac J. (2019). Comparative genomics of rumen Butyrivibrio spp. uncovers a continuum of polysaccharide-degrading capabilities. *Appl. Environ. Microbiol.* 86:e01993-19. 10.1128/AEM.01993-19 31653790 PMC6912079

[B47] Pan S. J. Cheung W. L. Fung H. K. Floudas C. A. Link A. J. (2010). Computational design of the lasso peptide antibiotic microcin J25. *Protein Eng. Des. Sel.* 24 275–282. 10.1093/protein/gzq108 21106549 PMC3038460

[B48] Potterat O. Stephan H. Metzger J. W. Gnau V. Zähner H. Jung G. (1994). Aborycin–A tricyclic 21-peptide antibiotic isolated from *Streptomyces griseoflavus*. *Justus Liebigs Ann. Chem.* 1994 741–743. 10.1002/jlac.199419940716

[B49] Potterat O. Wagner K. Gemmecker G. Mack J. Puder C. Vettermann R. (2004). BI-32169, a bicyclic 19-peptide with strong glucagon receptor antagonist activity from Streptomyces sp. *J. Nat. Prod.* 67 1528–1531. 10.1021/np040093o 15387654

[B50] Price M. N. Dehal P. S. Arkin A. P. (2010). FastTree 2–approximately maximum-likelihood trees for large alignments. *PLoS One* 5:e9490. 10.1371/journal.pone.0009490 20224823 PMC2835736

[B51] Rosengren K. J. Clark R. J. Daly N. L. Göransson U. Jones A. Craik D. J. (2003). Microcin J25 has a threaded sidechain-to-backbone ring structure and not a head-to-tail cyclized backbone. *J. Am. Chem. Soc.* 125 12464–12474. 10.1021/ja0367703 14531690

[B52] Russell J. B. Mantovani H. C. (2002). The bacteriocins of ruminal bacteria and their potential as an alternative to antibiotics. *J. Mol. Microbiol. Biotechnol.* 4 347–355. 12125815

[B53] Salomon R. A. Farías R. N. (1992). Microcin 25, a novel antimicrobial peptide produced by *Escherichia coli*. *J. Bacteriol.* 174 7428–7435. 10.1128/jb.174.22.7428-7435.1992 1429464 PMC207439

[B54] Seemann T. (2014). Prokka: rapid prokaryotic genome annotation. *Bioinformatics* 30 2068–2069. 10.1093/bioinformatics/btu153 24642063

[B55] Seshadri R. Leahy S. C. Attwood G. T. Teh K. H. Lambie S. C. Cookson A. L. (2018). Cultivation and sequencing of rumen microbiome members from the Hungate1000 Collection. *Nat. Biotechnol.* 36 359–367. 10.1038/nbt.4110 29553575 PMC6118326

[B56] Severinov K. Semenova E. Kazakov A. Kazakov T. Gelfand M. S. (2007). Low-molecular-weight post-translationally modified microcins. *Mol. Microbiol.* 65 1380–1394. 10.1111/j.1365-2958.2007.05874.x 17711420

[B57] Sievers F. Wilm A. Dineen D. Gibson T. J. Karplus K. Li W. (2011). Fast, scalable generation of high-quality protein multiple sequence alignments using Clustal Omega. *Mol. Syst. Biol.* 7:539. 10.1038/msb.2011.75 21988835 PMC3261699

[B58] Solbiati J. O. Ciaccio M. Farías R. N. González-Pastor J. E. Moreno F. Salomón R. A. (1999). Sequence analysis of the four plasmid genes required to produce the circular peptide antibiotic microcin J25. *J. Bacteriol.* 181 2659–2662. 10.1128/JB.181.8.2659-2662 10198038 PMC93700

[B59] Solbiati J. O. Ciaccio M. Farias R. N. Salomón R. A. (1996). Genetic analysis of plasmid determinants for microcin J25 production and immunity. *J. Bacteriol.* 178 3661–3663. 10.1128/jb.178.12.3661-3663 8655570 PMC178142

[B60] Soudy R. Wang L. Kaur K. (2012). Synthetic peptides derived from the sequence of a lasso peptide microcin J25 show antibacterial activity. *Bioorg. Med. Chem.* 20 1794–1800. 10.1016/j.bmc.2011.12.061 22304849

[B61] Steele D. B. Stowers M. D. (1991). Techniques for selection of industrially important microorganisms. *Annu. Rev. Microbiol.* 45 89–106. 10.1146/annurev.mi.45.100191.000513 1741626

[B62] Sumida T. Dubiley S. Wilcox B. Severinov K. Tagami S. (2019). Structural basis of leader peptide recognition in lasso peptide biosynthesis pathway. *ACS Chem. Biol.* 14 1619–1627. 10.1021/acschembio.9b00348 31188556

[B63] Tietz J. I. Schwalen C. J. Patel P. S. Maxson T. Blair P. M. Tai H.-C. (2017). A new genome-mining tool redefines the lasso peptide biosynthetic landscape. *Nat. Chem. Biol.* 13 470–478. 10.1038/nchembio.2319 28244986 PMC5391289

[B64] Trmcic A. Monnet C. Rogelj I. Bogovic Matijasic B. (2011). Expression of nisin genes in cheese–a quantitative real-time polymerase chain reaction approach. *J. Dairy Sci.* 94 77–85. 10.3168/jds.2010-3677 21183019

[B65] Tsunakawa M. Hu S.-L. Hoshino Y. Detlefson D. J. Hill S. E. Furumai T. (1995). Siamycins I and II, new anti-HIV peptides: I. Fermentation, isolation, biological activity and initial characterization. *J. Antibiot.* 48 433–434. 10.7164/antibiotics.48.433 7797448

[B66] van Heel A. J. de Jong A. Song C. Viel J. H. Kok J. Kuipers O. P. (2018). BAGEL4: a user-friendly web server to thoroughly mine RiPPs and bacteriocins. *Nucleic Acids Res.* 46 W278–W281. 10.1093/nar/gky383 29788290 PMC6030817

[B67] Weber T. Kim H. U. (2016). The secondary metabolite bioinformatics portal: computational tools to facilitate synthetic biology of secondary metabolite production. *Synth. Syst. Biotechnol.* 1 69–79. 10.1016/j.synbio.2015.12.002 29062930 PMC5640684

[B68] Weber W. Fischli W. Hochuli E. Kupfer E. Weibel E. K. (1991). Anantin-a peptide antagonist of the atrial natriuretic factor (ANF). I. Producing organism, fermentation, isolation and biological activity. *J. Antibiot.* 44 164–171. 10.7164/antibiotics.44.164 1849131

[B69] Winter J. M. Behnken S. Hertweck C. (2011). Genomics-inspired discovery of natural products. *Curr. Opin. Chem. Biol.* 15 22–31. 10.1016/j.cbpa.2010.10.020 21111667

[B70] Yan K.-P. Li Y. Zirah S. Goulard C. Knappe T. A. Marahiel M. A. (2012). Dissecting the maturation steps of the lasso peptide microcin J25 in vitro. *ChemBioChem* 13 1046–1052. 10.1002/cbic.201200016 22488892

[B71] Yano K. Toki S. Nakanishi S. Ochiai K. Ando K. Yoshida M. (1996). MS-271, a novel inhibitor of calmodulin-activated myosin light chain kinase from Streptomyces sp.—I. isolation, structural determination and biological properties of MS-271. *Bioorg. Med. Chem.* 4 115–120. 10.1016/0968-0896(95)00175-1 8689231

[B72] Zhu S. Fage C. D. Hegemann J. D. Mielcarek A. Yan D. Linne U. (2016a). The B1 protein guides the biosynthesis of a lasso peptide. *Sci. Rep.* 6:35604. 10.1038/srep35604 27752134 PMC5067487

[B73] Zhu S. Fage C. D. Hegemann J. D. Yan D. Marahiel M. A. (2016b). Dual substrate-controlled kinase activity leads to polyphosphorylated lasso peptides. *FEBS Lett.* 590 3323–3334. 10.1002/1873-3468.12386 27585551

[B74] Zhu S. Hegemann J. D. Fage C. D. Zimmermann M. Xie X. Linne U. (2016c). Insights into the unique phosphorylation of the lasso peptide paeninodin. *J. Biol. Chem.* 291 13662–13678. 10.1074/jbc.M116.722108 27151214 PMC4919450

[B75] Zong C. Cheung-Lee W. L. Elashal H. E. Raj M. Link A. J. (2018). Albusnodin: an acetylated lasso peptide from Streptomyces albus. *ChemComm.* 54 1339–1342. 10.1039/C7CC08620B 29350227 PMC5835390

[B76] Zyubko T. Serebryakova M. Andreeva J. Metelev M. Lippens G. Dubiley S. (2019). Efficient in vivo synthesis of lasso peptide pseudomycoidin proceeds in the absence of both the leader and the leader peptidase. *Chem. Sci.* 10 9699–9707. 10.1039/C9SC02370D 32055339 PMC6993621

