# Supplementary material for: In silico Screening Unveil the Great Potential of Ruminal Bacteria Synthesizing Lasso Peptides
Source: Front Microbiol. 2020 Sep 11;11:576738. doi: 10.3389/fmicb.2020.576738 (PMC7533575; doi:10.3389/fmicb.2020.576738)
Supplement: Supplementary file 1 [file Data_Sheet_1.pdf]

Supplementary Figures

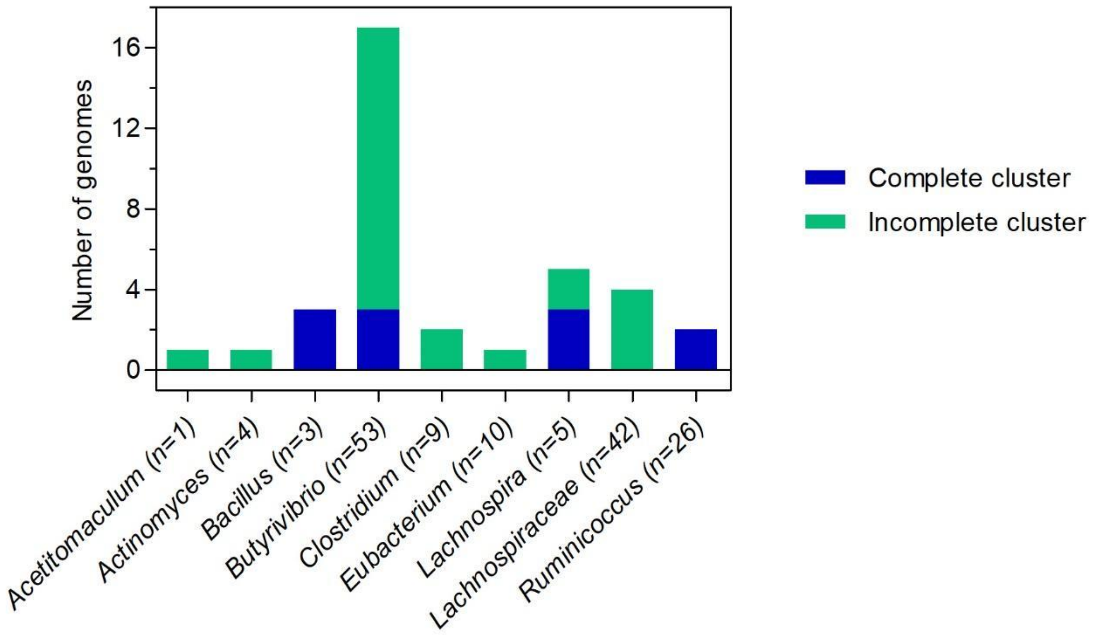

Figure S1. The number of genomes in different genera of ruminal bacteria showing predicted lasso peptide biosynthetic gene clusters. Complete clusters represent genomes containing the essential genes (*lasA*, *lasB*, and *lasC*) required for lasso peptide biosynthesis, while incomplete clusters contain at least one of these genes.

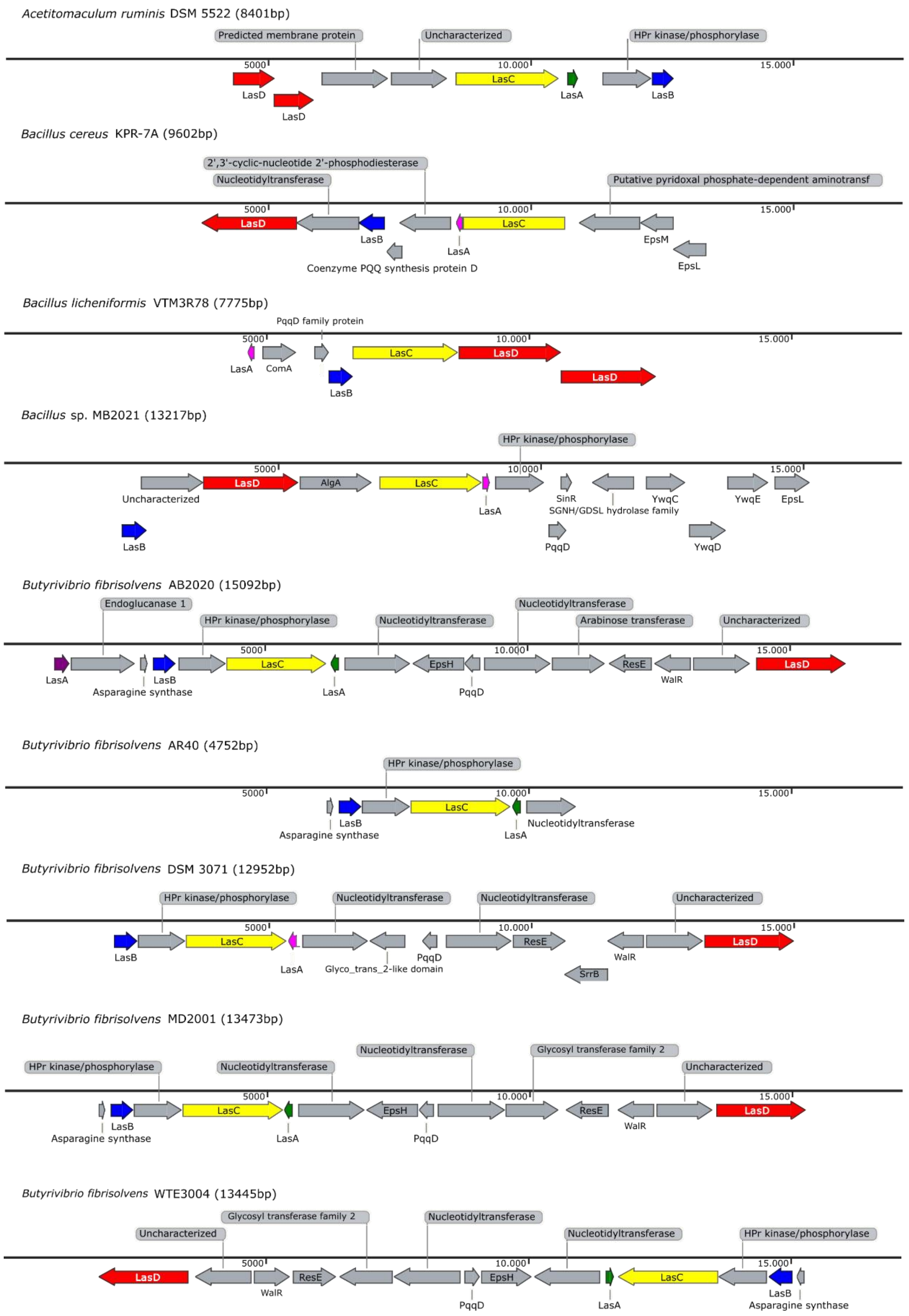
Acetitomaculum ruminis DSM 5522 (8401bp)
Predicted membrane protein
Uncharacterized
HPr kinase/phosphorylase
LasD
LasC
LasA
LasB
Bacillus cereus KPR-7A (9602bp)
2',3'-cyclic-nucleotide 2'-phosphodiesterase
Nucleotidyltransferase
Putative pyridoxal phosphate-dependent aminotransf
Coenzyme PQQ synthesis protein D
EpsM
EpsL
Bacillus licheniformis VTM3R78 (7775bp)
PqqD family protein
ComA
Bacillus sp. MB2021 (13217bp)
AlgA
SinR
SGNH/GDSL hydrolase family
YwqC
YwqE
EpsL
PqqD
YwqD
Butyrivibrio fibrisolvens AB2020 (15092bp)
Endoglucanase 1
Arabinose transferase
Asparagine synthase
EpsH
ResE
WalR
Butyrivibrio fibrisolvens AR40 (4752bp)
Butyrivibrio fibrisolvens DSM 3071 (12952bp)
Glyco_trans_2-like domain
SrrB
Butyrivibrio fibrisolvens MD2001 (13473bp)
Glycosyl transferase family 2
Butyrivibrio fibrisolvens WTE3004 (13445bp)
5000
10.000
15.000

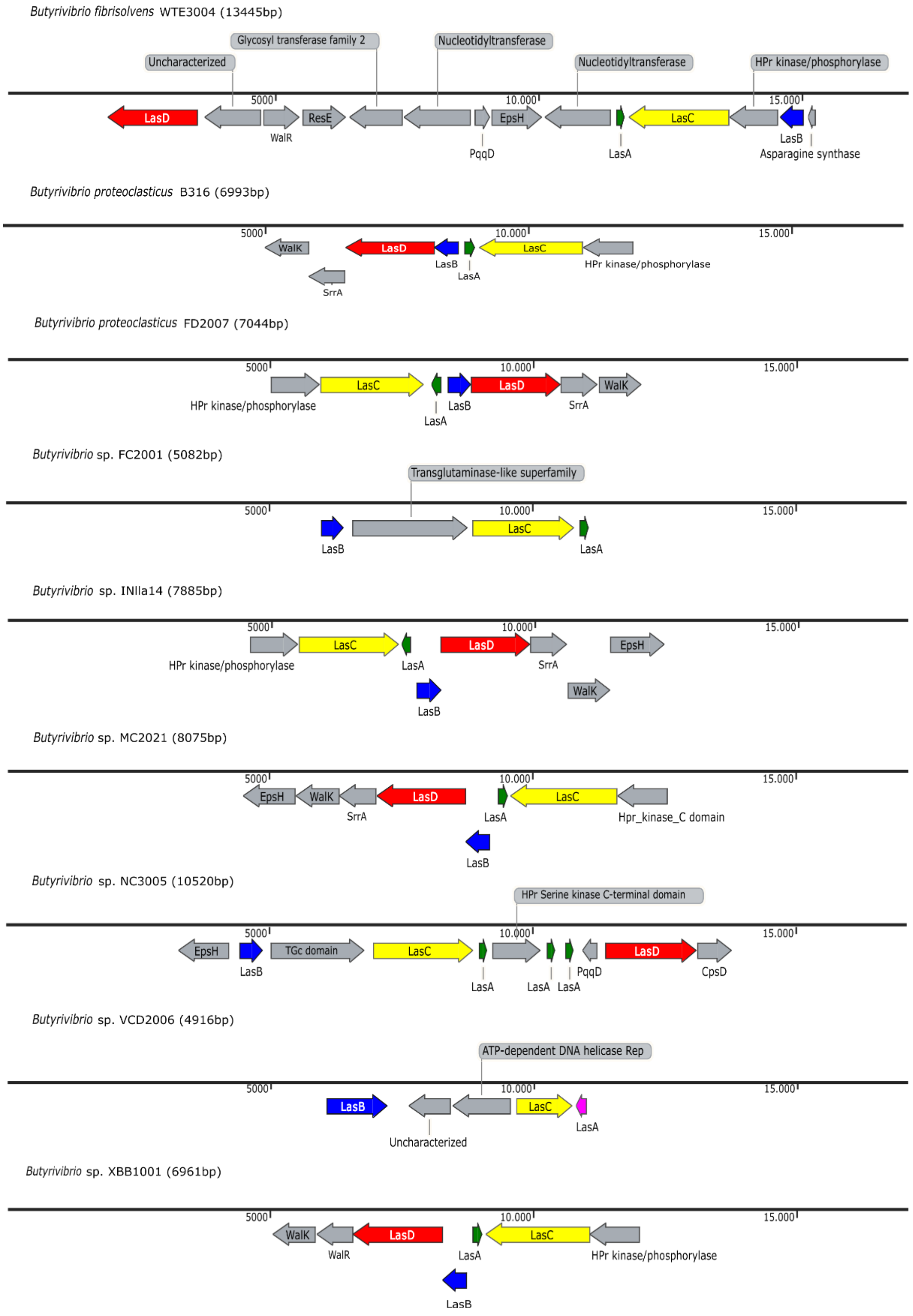
Butyrivibrio fibrisolvens WTE3004 (13445bp)
Glycosyl transferase family 2
Nucleotidyltransferase
Uncharacterized
Nucleotidyltransferase
HPr kinase/phosphorylase
5000
10.000
15.000
LasD
ResE
EpsH
LasC
WalR
PqqD
LasA
LasB
Asparagine synthase
Butyrivibrio proteoclasticus B316 (6993bp)
5000
10.000
15.000
WalK
LasD
LasC
LasB
LasA
HPr kinase/phosphorylase
SrrA
Butyrivibrio proteoclasticus FD2007 (7044bp)
5000
10.000
15.000
LasC
LasD
WalK
HPr kinase/phosphorylase
LasB
SrrA
LasA
Butyrivibrio sp. FC2001 (5082bp)
Transglutaminase-like superfamily
5000
10.000
15.000
LasC
LasB
LasA
Butyrivibrio sp. INIIa14 (7885bp)
5000
10.000
15.000
LasC
LasD
EpsH
HPr kinase/phosphorylase
LasA
SrrA
WalK
LasB
Butyrivibrio sp. MC2021 (8075bp)
5000
10.000
15.000
EpsH
WalK
LasD
LasC
SrrA
LasA
Hpr_kinase_C domain
LasB
Butyrivibrio sp. NC3005 (10520bp)
HPr Serine kinase C-terminal domain
5000
10.000
15.000
EpsH
TGc domain
LasC
LasD
LasB
PqqD
CpsD
LasA
LasA
LasA
Butyrivibrio sp. VCD2006 (4916bp)
ATP-dependent DNA helicase Rep
5000
10.000
LasB
LasC
LasA
Uncharacterized
Butyrivibrio sp. XBB1001 (6961bp)
5000
10.000
15.000
WalK
LasD
LasC
WalR
LasA
HPr kinase/phosphorylase
LasB

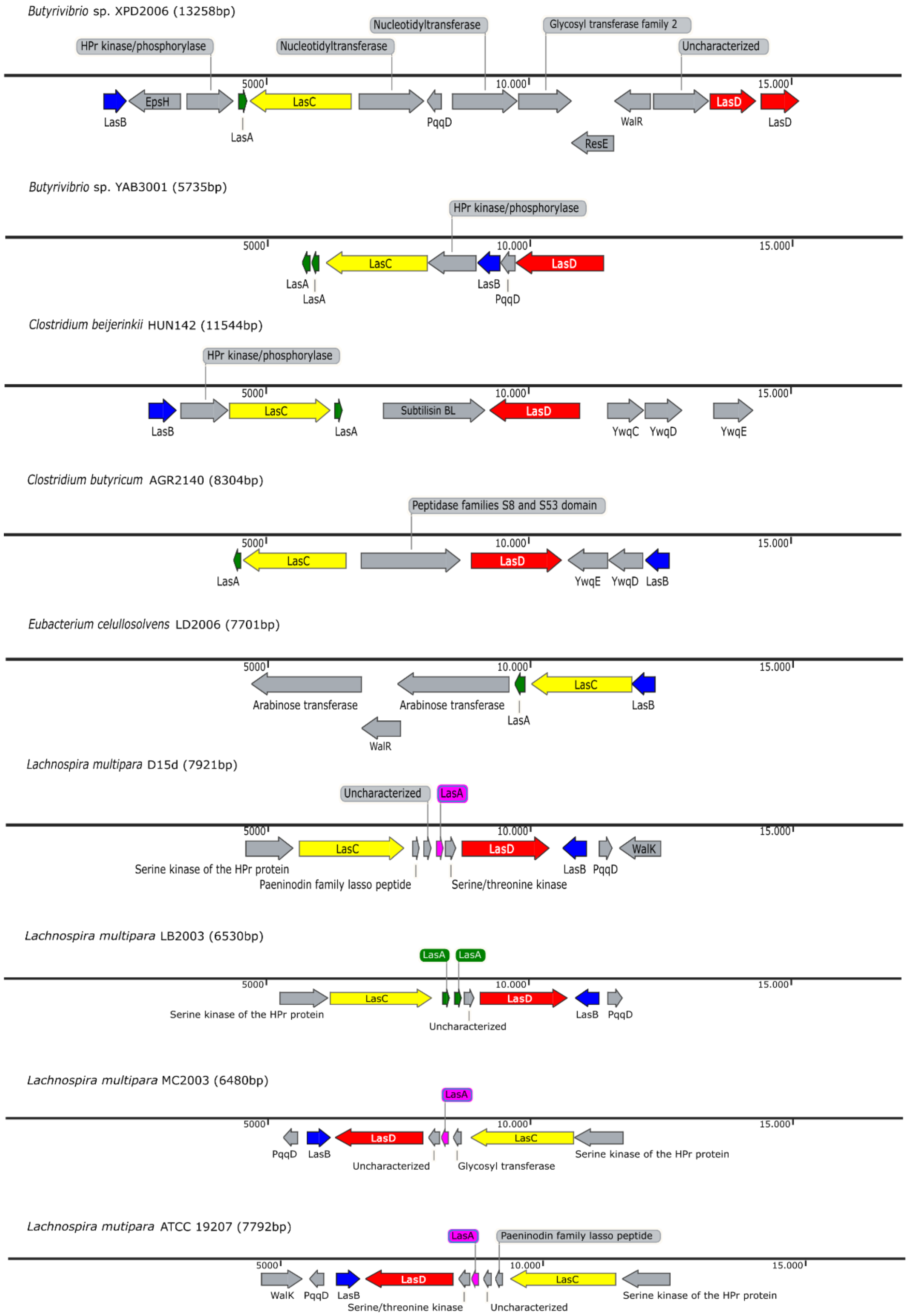

Butyrivibrio sp. XPD2006 (13258bp)
Nucleotidyltransferase
Glycosyl transferase family 2
HPr kinase/phosphorylase
Nucleotidyltransferase
Uncharacterized
5000
10.000
15.000
EpsH
LasC
LasD
LasB
LasA
PqqD
WalR
LasD
ResE
Butyrivibrio sp. YAB3001 (5735bp)
HPr kinase/phosphorylase
5000
10.000
15.000
LasC
LasD
LasA
LasA
LasB
PqqD
Clostridium beijerinkii HUN142 (11544bp)
HPr kinase/phosphorylase
5000
10.000
15.000
LasC
Subtilisin BL
LasD
LasB
LasA
YwqC
YwqD
YwqE
Clostridium butyricum AGR2140 (8304bp)
Peptidase families S8 and S53 domain
5000
10.000
15.000
LasC
LasD
LasA
YwqE
YwqD
LasB
Eubacterium celullosolvens LD2006 (7701bp)
5000
10.000
15.000
LasC
Arabinose transferase
Arabinose transferase
LasB
LasA
WalR
Lachnospira multipara D15d (7921bp)
Uncharacterized
LasA
5000
10.000
15.000
LasC
LasD
WalK
Serine kinase of the HPr protein
Paeninodin family lasso peptide
Serine/threonine kinase
LasB
PqqD
Lachnospira multipara LB2003 (6530bp)
LasA
LasA
5000
10.000
15.000
LasC
LasD
Serine kinase of the HPr protein
Uncharacterized
LasB
PqqD
Lachnospira multipara MC2003 (6480bp)
LasA
5000
10.000
15.000
LasD
LasC
PqqD
LasB
Uncharacterized
Glycosyl transferase
Serine kinase of the HPr protein
Lachnospira mutipara ATCC 19207 (7792bp)
LasA
Paeninodin family lasso peptide
5000
10.000
15.000
LasD
LasC
WalK
PqqD
LasB
Serine/threonine kinase
Uncharacterized
Serine kinase of the HPr protein

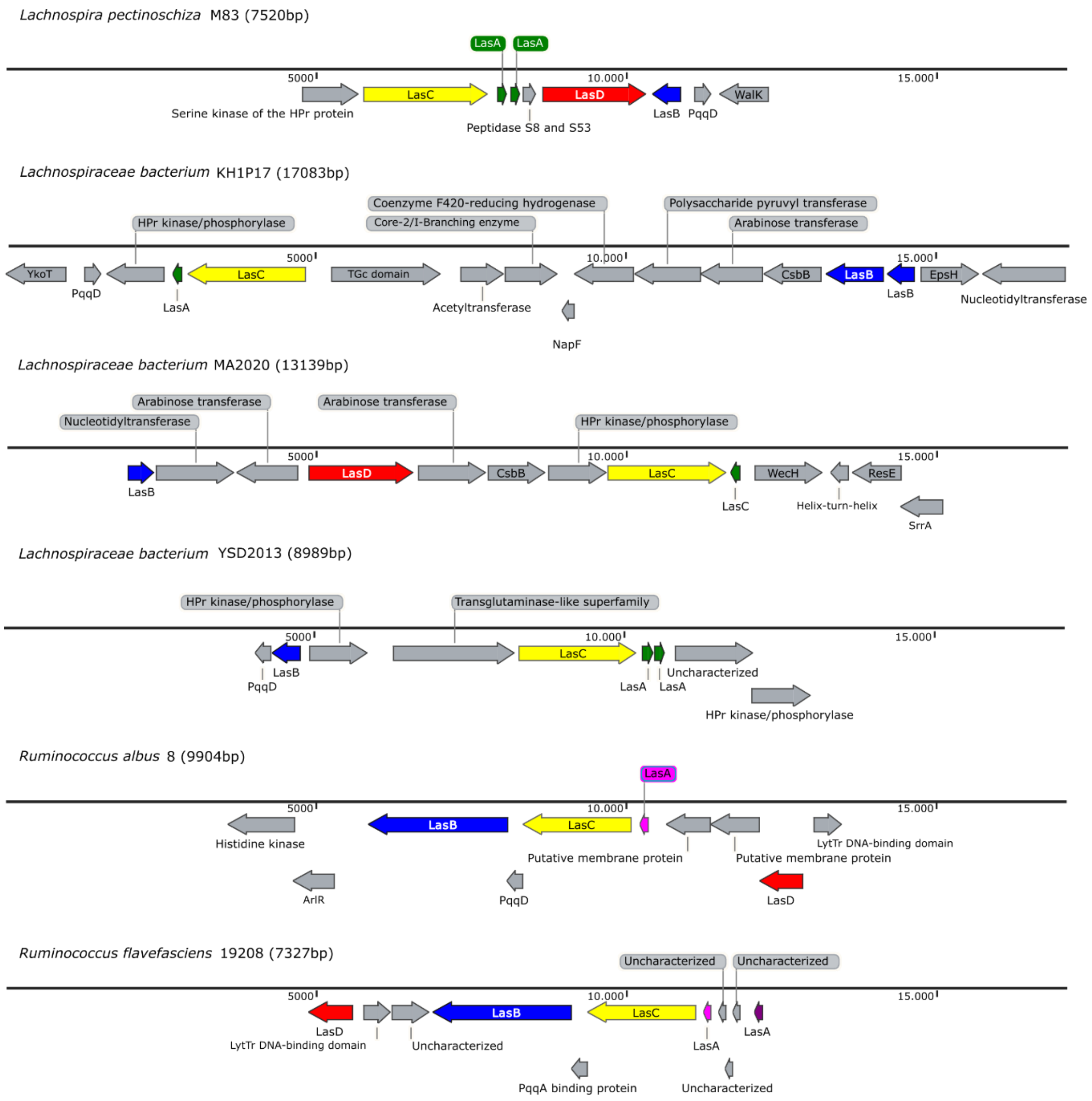

Figure S2. Schematic showing the genomic context of the lasso peptide gene clusters in the genomes of ruminal bacterial. The genes encoding putative LasA precursors were found by manual curation of the genomic context (green), and genome mining using antiSMASH (pink) and BAGEL4 (purple). Genes encoding the putative maturation enzymes LasB (blue) and LasC (yellow), and the ABC-transporter LasD (red) are also shown in the biosynthetic cluster. Additional genes found within or in the vicinity of the lasso peptide gene cluster are represented in gray. The genetic context representation was constructed using SnapGene Viewer 5.0.8.

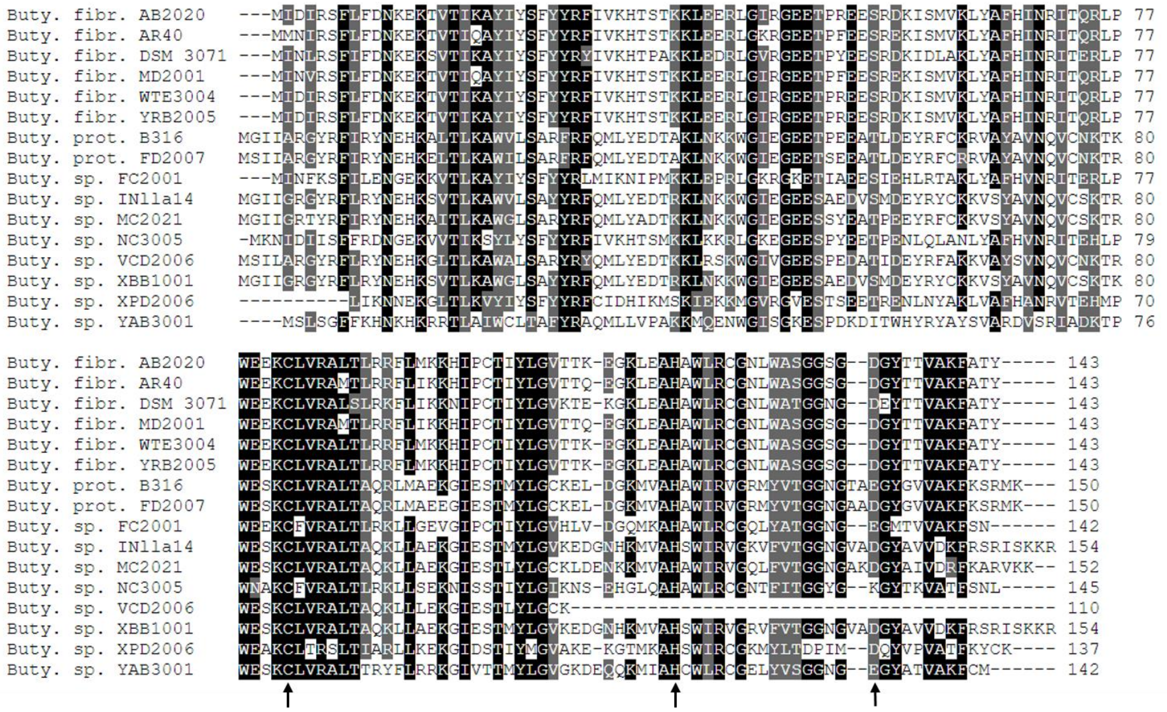

Figure S3. Alignment of the putative LasB proteins from strains of ruminal *Butyrivibrio*. The alignment was performed using Clustal Omega 1.2.3 and the conserved sequences were colored using Color Align Conservation (https://www.bioinformatics.org/sms2/color_align_cons.html) setting the similarity criteria at 70%. Black arrows indicate the conserved Cys-His-Asp catalytic triad in the predicted active site of the protein.

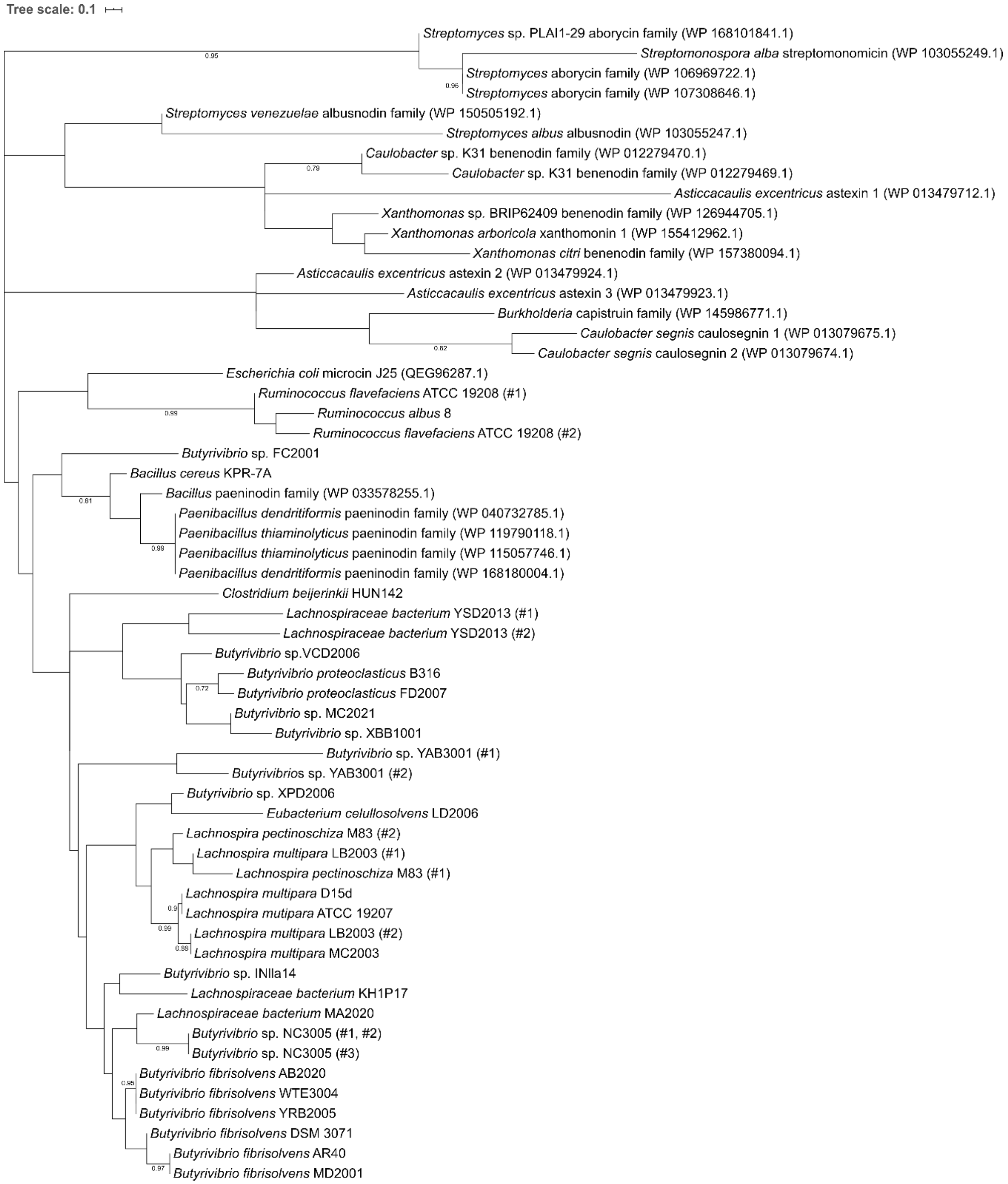

Figure S4. Phylogenetic tree of known lasso peptides and the new LasA-like peptides predicted in the current study. The sequences of lasso peptides that were reported previously were obtained from NCBI and the accession numbers are presented in front of the organism name. The alignment was performed using Muscle and the tree was generated using the Maximum-Likelihood method with 100 replicates using Mega 7.0.26. Only bootstrap values greater than 70% are shown at the nodal branches.
